# INTERNATIONAL KIDNEY CANCER SYMPOSIUM: NORTH AMERICA

**DOI:** 10.15586/jkcvhl.v13iS1.457

**Published:** 2026-03-16

**LEAD AUTHOR**

Cappello, Angela, University of Bari, Department of Interdisciplinary Medicine, Laboratory of Molecular Oncology & Division of Medical Oncology, A.O.U. Consorziale Policlinico di Bari, Bari, Italy

**CO-AUTHORS**

Giuseppina Leuzzi, University of Bari, Department of Interdisciplinary Medicine, Laboratory of Molecular Oncology & Division of Medical Oncology, A.O.U. Consorziale Policlinico di Bari, Bari, Italy

Sara Di Bari, University of Bari, Department of Interdisciplinary Medicine, Laboratory of Molecular Oncology & Division of Medical Oncology, A.O.U. Consorziale Policlinico di Bari, Bari, Italy

Lavinia Ferrone, University of Bari, Department of Interdisciplinary Medicine, Laboratory of Molecular Oncology & Division of Medical Oncology, A.O.U. Consorziale Policlinico di Bari, Bari, Italy

Carlo Ganini, University of Bari, Department of Interdisciplinary Medicine, Laboratory of Molecular Oncology & Division of Medical Oncology, A.O.U. Consorziale Policlinico di Bari, Bari, Italy

Camillo Porta, University of Bari, Department of Interdisciplinary Medicine, Laboratory of Molecular Oncology & Division of Medical Oncology, A.O.U. Consorziale Policlinico di Bari, Bari, Italy

Clear cell Renal Cell Carcinoma (ccRCC) is the prevalent subtype of kidney cancer characterized by mutations in the Von Hippel Lindau (VHL) gene, leading to dysregulated hypoxia signaling. While VHL mutations contribute to tumor initiation, additional genetic and epigenetic events are required for cancer progression. Dysregulated amino acid metabolism, particularly leucine catabolism mediated by the enzyme 3-Methylglutaconyl-CoA hydratase, also called AUH-AU RNA binding protein, has emerged as a critical factor in the development of ccRCC metastasis and aggressiveness. Current therapeutic strategies for ccRCC, including angiogenesis inhibitors and immunotherapy, often encounter resistance, highlighting the need to understand the underlying molecular mechanisms. Emerging evidence implicates amino acid metabolism, particularly leucine catabolism via the mitochondrial enzyme AUH, as a critical modulator of ccRCC progression and therapeutic response. In this study, we investigate the molecular and metabolic consequences of AUH loss in ccRCC, with a focus on the development of possible therapeutic resistance mechanisms. Bioinformatic analysis of public datasets (cBioPortal) revealed that AUH is down-expressed in approximately 64% of renal cancer cases, with this alteration correlating with poor prognosis. Moreover, public ChIP-seq data suggest that AUH expression may be under the regulatory control of HIF proteins. While hypoxia and CoCl_2_ treatment induce AUH mRNA expression, its protein levels decrease, implying post-transcriptional repression. Moreover, AUH silencing in ccRCC cells leads to reduced Reactive Oxygen Species (ROS) production by modulating electron transport chain protein expression, lipid droplet accumulation, and upregulation of GPX4. These data suggest a potential growth advantage in ccRCC after decreased AUH expression, which is also mediated by enhanced tumor stemness (spheroid formation), making the pharmacological manipulation of leucine metabolism an appealing therapeutical target to be further explored in ccRCC.

