# Supplementary material for: INTERNATIONAL KIDNEY CANCER SYMPOSIUM: NORTH AMERICA
Source: J Kidney Cancer VHL. 2026 Mar 16;13(SP1):7–65. doi: 10.15586/jkcvhl.v13iS1.457 (PMC13660973; doi:10.15586/jkcvhl.v13iS1.457)
Supplement: Related texts

# Abstract: BAP1 Deficiency Enhances Spheroid Formation and Metabolic Flexibility in ccRCC via IP3R3 Regulation

**LEAD AUTHOR**

Ganini, Carlo, University of Bari, Department of Interdisciplinary Medicine, Laboratory of Molecular Oncology & Azienda Ospedaliero Universitaria Consorziale Policlinico di Bari, Bari, Italy

**CO-AUTHORS**

Giuseppina Leuzzi, University of Bari, Department of Interdisciplinary Medicine, Laboratory of Molecular Oncology & Azienda Ospedaliero Universitaria Consorziale Policlinico di Bari, Bari, Italy

Angela Cappello, University of Bari, Department of Interdisciplinary Medicine, Laboratory of Molecular Oncology & Azienda Ospedaliero Universitaria Consorziale Policlinico di Bari, Bari, Italy

Lavinia Ferrone, University of Bari, Department of Interdisciplinary Medicine, Laboratory of Molecular Oncology & Azienda Ospedaliero Universitaria Consorziale Policlinico di Bari, Bari, Italy

Sara Di Bari, University of Bari, Department of Interdisciplinary Medicine, Laboratory of Molecular Oncology & Azienda Ospedaliero Universitaria Consorziale Policlinico di Bari, Bari, Italy

Camillo Porta, University of Bari, Department of Interdisciplinary Medicine, Laboratory of Molecular Oncology & Azienda Ospedaliero Universitaria Consorziale Policlinico di Bari, Bari, Italy

BRCA1-associated protein 1 (BAP1) is a tumor suppressor involved in crucial cellular processes including DNA repair, chromatin remodeling, and calcium signaling. Its loss has been associated with poor prognosis in various cancers, including clear cell renal cell carcinoma (ccRCC). This study investigates the functional role and prognostic significance of BAP1 in ccRCC, with a particular focus on its relationship with inositol 1,4,5-trisphosphate receptor type 3 (IP3R3), a regulator of mitochondrial calcium signaling. Using publicly available datasets (e.g., TCGA PanCancer Atlas), we analyzed BAP1 mutational status and gene expression across ccRCC samples. BAP1 loss was found to correlate with reduced mRNA expression of IP3R3 and increased expression of stemness markers such as CD44, MKI67, and ALDH1A1, suggesting a more aggressive tumor phenotype. Survival analyses indicated a trend toward poorer overall survival in patients with low BAP1 or IP3R3 expression, although not statistically significant. In vitro experiments in ccRCC cell lines (786-O and CAKI-1) demonstrated that knockdown of BAP1 or IP3R3 leads to an increased number of spheroids, reflecting enhanced clonogenic capacity. Notably, cell viability, apoptosis, and doubling time were not significantly affected by knockdown of either gene. However, metabolic stress assays using glycolysis and oxidative phosphorylation inhibitors (e.g., 2-deoxy-D-glucose, oligomycin, rotenone) revealed that BAP1 or IP3R3 loss may enhance cell adaptability under nutrient-limiting conditions, suggesting a role in metabolic reprogramming. Additional transcriptomic data support the involvement of BAP1 in modulating cellular plasticity and mitochondrial homeostasis. Together, these findings highlight a potential mechanistic link between BAP1 loss, altered mitochondrial calcium signaling via IP3R3, and acquisition of stem-like features in ccRCC. While not yet definitive as prognostic markers, BAP1 and IP3R3 represent promising molecular targets for further therapeutic investigation in renal cancer.

# Abstract: A multimodal comparison of metabolic profiling methods to explore tumor heterogeneity with single-cell resolution

**LEAD AUTHOR**

Patankar, Madhura, UC Davis

**CO-AUTHORS**

Elijah Kofke, UC Davis

Michael Pargett, UC Davis

Daniel Oberbauer, UC Davis

Chin-Hsien Chen, UC Davis

John Albeck, UC Davis

Shuchi Gulati, UC Davis

Background: Metabolic dysregulation is a hallmark of oncogenesis. A classic example is the heightened preference for aerobic glycolysis—the Warburg effect—which is especially prevalent in kidney cancers due to VHL mutation–driven HIF upregulation. While methods such as isotope labeling, metabolite readers, Seahorse assays, and SCENITH profiling capture diverse metabolic parameters, they rely on bulk sampling, obscuring single-cell variability and spatial context. Fluorescent biosensors like AMPKAR (AMPK activity reporter) and HYlight (fructose 1,6-bisphosphate reporter) enable single-cell metabolic monitoring, yet linking these readouts to bulk measurements remains challenging.

Methods: We directly compared bulk and single-cell metabolic assays, including Seahorse, SCENITH, and biosensor-based methods. In addition, we integrated an imaging-adapted click-chemistry approach using O-propargyl puromycin (OPP) to quantify protein translation rates. This assay complements live-cell imaging and other single-cell techniques, allowing dynamic, spatially resolved measurement of metabolic activity.

Results: Integrated analysis of Seahorse data with quantitative live-cell imaging revealed that cultured renal carcinoma cells preferentially engage glycolysis over mitochondrial respiration. Inhibition of glycolysis with 2-deoxy-D-glucose (2DG) significantly reduced translational output, confirming functional consequences of altered metabolic states. The imaging-adapted translation assay provided complementary insights to Seahorse and SCENITH, linking changes in energy metabolism to translational capacity at the single-cell level. Additionally, we found that certain inhibitors of signaling induced rapid but heterogeneous decreases in glycolytic indicators such as fructose 1,6-bisphosphate.

Conclusion: Combining bulk and multidimensional single-cell analyses addresses a key gap in metabolic profiling by resolving cellular heterogeneity. This approach will be essential for understanding tumor–microenvironment metabolic interactions and for screening candidate inhibitors of metabolism to identify patient-tailored cancer treatments. This framework also offers potential for profiling non-oncogenic cells in the tumor microenvironment, such as TME infiltrating immune cells, providing functional characterization to guide therapeutic optimization.



# Abstract: TRIP13 is upregulated in aggressive chromophobe renal cell carcinoma and may be a promising therapeutic target

**LEAD AUTHOR**

Ellis, Elizabeth, University of Texas MD Anderson Cancer Center

**CO-AUTHORS**

Nathan Maulding, Tempus AI

Keiko Akagi, University of Texas MD Anderson Cancer Center

Zhiyuan Yu, University of Texas MD Anderson Cancer Center

Menuka Karki, University of Texas MD Anderson Cancer Center

Nicholas Scott, University of Texas MD Anderson Cancer Center

Pedro Pesquera, University of Texas MD Anderson Cancer Center

Jing Qian, University of Texas MD Anderson Cancer Center

Fei Duan, University of Texas MD Anderson Cancer Center

Abha Grover, University of Texas MD Anderson Cancer Center

Kyle Beauchamp, Tempus AI

Kaveh Karbasi, University of Texas MD Anderson Cancer Center

Kanishka Sircar, University of Texas MD Anderson Cancer Center

Luisa Maren Solis Soto, University of Texas MD Anderson Cancer Center

Xinyue Chen, University of Texas MD Anderson Cancer Center

Andrew Sedgewick, Tempus AI Justin Guinney, Tempus AI

Jose Karam, University of Texas MD Anderson Cancer Center

Pavlos Msaouel, University of Texas MD Anderson Cancer Center

Niki Zacharias, University of Texas MD Anderson Cancer Center

Chromophobe renal cell carcinoma (chRCC) with sarcomatoid features is one of the most lethal renal malignancies. We identified a protein critical to the metastatic potential of chRCC. We describe our experience in preclinical models inhibiting this protein, TRIP13.

Targeted DNA profiling, whole transcriptome gene expression and de-identified clinical data from 168 chRCC samples were collected. Whole-exome and RNA sequencing were performed in a sarcomatoid chRCC tumor and corresponding patient-derived xenograft (RCC251). TRIP13 expression was determined using immunohistochemistry. We performed knockdown of TRIP13 (KD) in UOK276 cells using lentiviral transfection using a vector corresponding to 3 siRNAs against TRIP13 and scramble vector as a control (SC). Scratch, 24-hour invasion, and clonogenic assays were performed in triplicate on stably transfected cells. Wildtype UOK276 cells were injected subcutaneously into NSG mice. Mice were treated with TRIP13 inhibitor DCZ0415 25 mg/kg IP thrice weekly (n = 8) or vehicle (n = 7).

TP53 mutations were found in 76 of 168 chRCC patient samples (45%). TP53 mutants had higher expression of TRIP13 than TP53 WT tumors (p < 1 x 10-10). Transcript expression of TRIP13 was 8-fold higher in PDX tumor compared to the original patient’s normal kidney and non-sarcomatoid regions. TRIP13 was enriched in samples that had ≥6 copy-neutral-loss-of-heterozygosity (cnLOH) events compared to those with <6 cnLOH events (p < 1 × 10-5). Cell proliferation, migration, and invasion were reduced in TRIP13 KD cells compared to SC. Reduced tumor growth (p < 0.0001) and tumor mass (p = 0.005) was observed in tumor-bearing UOK276 mice treated with the TRIP13 inhibitor DCZ0415 compared to vehicle.

TRIP13 is upregulated in preclinical models of sarcomatoid chRCC and patient tumors. TRIP13 inhibition reduced tumor growth in a sarcomatoid chRCC animal model. TRIP13 may be a promising therapeutic target in aggressive sarcomatoid chRCC meriting further investigation.



# Abstract: Association of G-coupled protein receptor 65 (GPR65), a novel therapeutic target, with immunotherapy response in metastatic renal cell carcinoma (mRCC)

**LEAD AUTHOR**

Shah, Koral U., Department of Medical Oncology & Therapeutics Research, City of Hope Comprehensive Cancer Center, Duarte, CA

**CO-AUTHORS**

Nicholas J. Salgia, Department of Immunology, Roswell Park Comprehensive Cancer Center, Buffalo, NY

Sumeet Ambarkhane, Pathios Therapeutics Limited, Oxford, United Kingdom

Barbara Cipriani, Pathios Therapeutics Limited, Oxford, United Kingdom

Danish Memon, M:M Bio Limited, Oxford, United Kingdom

Maria-Jose Oliva Martin, Pathios Therapeutics Limited, Oxford, United Kingdom

Miguel Zugman, Department of Medical Oncology & Therapeutics Research, City of Hope Comprehensive Cancer Center, Duarte, CA

Salvador Jaime-Casas, Department of Medical Oncology & Therapeutics Research, City of Hope Comprehensive Cancer Center, Duarte, CA

Hedyeh Ebrahimi, Department of Medical Oncology & Therapeutics Research, City of Hope Comprehensive Cancer Center, Duarte, CA

Daniela V. Castro, Department of Medical Oncology & Therapeutics Research, City of Hope Comprehensive Cancer Center, Duarte, CA

Benjamin Mercier, Department of Medical Oncology & Therapeutics Research, City of Hope Comprehensive Cancer Center, Duarte, CA

Peter D. Zang, Department of Medical Oncology & Therapeutics Research, City of Hope Comprehensive Cancer Center, Duarte, CA

Regina Barragan-Carrillo, Department of Medical Oncology & Therapeutics Research, City of Hope Comprehensive Cancer Center, Duarte, CA

JoAnn Hsu, Department of Medical Oncology & Therapeutics Research, City of Hope Comprehensive Cancer Center, Duarte, CA

Nazli Dizman, The University of Texas MD Anderson Cancer Center, Houston, TX

Wesley Yip, Division of Urology and Urologic Oncology, Department of Surgery, City of Hope Comprehensive Cancer Center, Duarte, CA

Alexander Chehrazi-Raffle, Department of Medical Oncology & Therapeutics Research, City of Hope Comprehensive Cancer Center, Duarte, CA

Abhishek Tripathi, Department of Medical Oncology & Therapeutics Research, City of Hope Comprehensive Cancer Center, Duarte, CA

Sumanta K. Pal, Department of Medical Oncology & Therapeutics Research, City of Hope Comprehensive Cancer Center, Duarte, CA

GPR65 is a pH-sensing receptor expressed on immune cells which mediates immunosuppressive effects in the tumor microenvironment (TME). Antitumor activity with a novel GPR65 inhibitor in murine xenografts bearing MC38 tumors has been reported previously. Here, we evaluate the relationship between GPR65 expression, TME phenotype, immune checkpoint inhibitor (ICI) response in mRCC, and characterize the immunomodulatory activity of GPR65 inhibition in human RCC tumor extracts.

Transcript-per-million normalized gene expression count matrices and clinical metadata were accessed from mRCC patients enrolled in the IMmotion151, Javelin101, and HCRN-GU16-260 trials, and from The Cancer Genome Atlas clear cell RCC (TCGA KIRC). Patients were stratified into GPR65high and GPR65low groups based on median expression value within each cohort in each trial. In separate translational studies, primary RCC tumors were exposed to varying concentrations of GPR65 inhibitor, PTT-3213, with assessment of secreted cytokine levels in culture media.

Within TCGA KIRC, GPR65 expression was significantly enriched in tumors compared to matched normal kidney (p<0.0001) and increased with tumor stage (p<0.05). In HCRN-GU16-260, where patients received ICI monotherapy as 1st-line treatment, GPR65high patients experienced significantly improved PFS vs. GPR65low (HR=0.49; p=0.009). GPR65 expression was enriched in PDL1+ tumors within both Javelin101 and IMmotion151 (p<0.001). Non-negative Matrix Factorization clustering in IMmotion151 demonstrated enrichment of T-effector/proliferative programs in GPR65high (p<0.00001) whereas GPR65low tumors were enriched for complement/omega-oxidation (p<0.00001) and proliferative programs (p=0.0009). Exposure of primary RCC tumors to PTT-3213 reduced anti-inflammatory cytokines (e.g., IL-10) and increased chemoattractant cytokines (e.g., CCL3).

These findings suggest that GPR65 expression marks a distinct TME with immune infiltration, PD-L1 upregulation, and offers insights into potential combination strategies. Herein supporting an ongoing phase 1 trial of a GPR65 inhibitor in solid tumors including RCC (NCT06634849).

# Abstract: Higher ectonucleotide pyrophosphate/ phosphodiesterase 3 (ENPP3) expression in patients with metastatic renal cell carcinoma (mRCC) is associated with an angiogenic subtype

**LEAD AUTHOR**

Li, Yu Jun, Department of Medical Oncology & Therapeutics Research, City of Hope Comprehensive Cancer Center, Duarte, CA

**CO-AUTHORS**

Nicholas Salgia, Department of Immunology, Roswell Park Comprehensive Cancer Center, Buffalo, NY, USA

Vitor Abreu de Góes, Department of Medical Oncology & Therapeutics Research, City of Hope Comprehensive Cancer Center, Duarte, CA

Salvador Jaime-Casas, Department of Medical Oncology & Therapeutics Research, City of Hope Comprehensive Cancer Center, Duarte, CA

Miguel Zugman, Department of Medical Oncology & Therapeutics Research, City of Hope Comprehensive Cancer Center, Duarte, CA

Koral Shah, Department of Medical Oncology & Therapeutics Research, City of Hope Comprehensive Cancer Center, Duarte, CA

Daniela Castro, Department of Medical Oncology & Therapeutics Research, City of Hope Comprehensive Cancer Center, Duarte, CA

Xiaochen Li, Department of Medical Oncology & Therapeutics Research, City of Hope Comprehensive Cancer Center, Duarte, CA

Benjamin Mercier, Department of Medical Oncology & Therapeutics Research, City of Hope Comprehensive Cancer Center, Duarte, CA

Regina Barragan- Carrillo, Department of Medical Oncology & Therapeutics Research, City of Hope Comprehensive Cancer Center, Duarte, CA

JoAnn Hsu, Department of Medical Oncology & Therapeutics Research, City of Hope Comprehensive Cancer Center, Duarte, CA

Ruchi Agarwal, Department of Medical Oncology & Therapeutics Research, City of Hope Comprehensive Cancer Center, Duarte, CA

Hedyeh Ebrahimi, Department of Medical Oncology & Therapeutics Research, City of Hope Comprehensive Cancer Center, Duarte, CA

Alexander Chehrazi-Raffle, Department of Medical Oncology & Therapeutics Research, City of Hope Comprehensive Cancer Center, Duarte, CA

Sumanta Pal, Department of Medical Oncology & Therapeutics Research, City of Hope Comprehensive Cancer Center, Duarte, CA

Background: ENPP3 is a cell surface enzyme that plays a role in purinergic signaling and immune response modulation. Markedly increased expression of ENPP3 in tumor versus normal tissue has led to recognition of this moiety as an attractive target for drug development in renal cell carcinoma (RCC), with two ENPP3 x CD3 bispecific T-cell antibody studies ongoing (NCT06178614, NCT05433142). We explore associations between ENPP3 expression and clinical outcome in prospective studies of RCC as a means of discerning optimal combination strategies.

Methods: We utilized normalized gene expression count matrices (transcripts-per-million) and clinical metadata from 3 prospective, randomized trials (Javelin101, IMmotion151, and HCRN-GU16-260). Stratifying by median ENPP3 expression within each trial, we compared baseline clinicopathologic characteristics and progression-free survival (PFS) between treatment arms in these studies. The IMmotion151 genomic classifier, grouping patients into 7 clusters based on nonnegative matrix factorization (NMF; Motzer et al. Cancer Cell. 2020), was used to characterize the study population in the context of ENPP3 expression.

Results: ENPP3 expression was found to be higher in patients lacking sarcomatoid features (P= 1.23 x 10-12) and in patients with a favorable IMDC score (vs poor IMDC score; P<0.0001). ENPP3high status failed to distinguish PFS benefit with the addition of checkpoint inhibitor (CPI) in the Javelin101 (n=886) or IMmotion151 (n=823) trials nor did it distinguish between outcomes with 1st-line CPI in the HCRN-GU16-260 experience (n=123). When evaluating subsets based on non-nuclear matrix factorization (NMF) clusters within IMmotion151, ENPP3high tumors were more frequently characterized as NMF1 (angiogenic stromal; P=0.02) or NMF2 (angiogenic; P<0.00001).

Conclusions: RCC tumors with increased ENPP3 expression more likely cluster within an angiogenic subtype. Taken together with our other findings, this supports future study of combinatorial approaches with ENPP3-targeted drugs paired with VEGF-directed therapy.

# Abstract: Obesity Attenuates Mitochondrial Dysfunction in Human Clear Cell Renal Cell Carcinoma

**LEAD AUTHOR**

Opurum, Precious, Diabetes & Metabolism Research Center, University of Utah, Salt Lake City, Utah, USA. 2 Department of Nutrition and Integrative Physiology, University of Utah, Salt Lake City, Utah, USA.

**CO-AUTHORS**

Cornelia Ulrich, Huntsman Cancer Institute, Cancer Hospital, Salt Lake City, Utah, USA.

Kyle Harshani, Department of Surgery, Division of Urology, University of Utah School of Medicine, Salt Lake City, Utah, USA.

Gayatri Khanderao, Department of Surgery, Division of Urology, University of Utah School of Medicine, Salt Lake City, Utah, USA.

Alejandro Sanchez,, Huntsman Cancer Institute, Cancer Hospital, Salt Lake City, Utah, USA. ;Department of Surgery, Division of Urology, University of Utah School of Medicine, Salt Lake City, Utah, USA.

Katsuhiko Funai, Diabetes & Metabolism Research Center, University of Utah, Salt Lake City, Utah, USA. ;Department of Nutrition and Integrative Physiology, University of Utah, Salt Lake City, Utah, USA; Department of Physical Therapy & Athletic Training, University of Utah

Clear cell renal cell carcinoma (ccRCC) is the most common and metabolically distinct subtype of kidney cancer. Despite obesity being the leading risk factor for ccRCC development, obese patients paradoxically experienced improved survival compared to their lean counterparts. The mechanisms underlying this obesity paradox remain poorly understood but may involve distinct metabolic programming in the tumor microenvironment, including mitochondrial dysfunction.

To investigate this, we assessed mitochondrial bioenergetics in n=# patients with ccRCC who underwent nephrectomy. We prospectively collected fresh tumors and matched normal kidney tissues at the time of surgery. Patients were stratified as obese (n=#, body mass index, BMI, ≥30 kg/m2) or normal/overweight (n=#, BMI <30 kg/m2) and normal/overweight (n=#, BMI <30 kg/m2). Using high-resolution respirometry and fluorimetry, we measured mitochondrial respiration, fatty acid oxidation (FAO), and ATP production. Transmission electron microscopy (TEM) was used to evaluate mitochondrial ultrastructure.

Tumors exhibited impaired mitochondrial respiration and ATP production compared to normal tissue, consistent with ccRCC’s well-documented metabolic dysfunction. Notably, tumors from obese patients showed partial preservation of mitochondrial function, with higher FAO rates and ADP-stimulated respiration relative to tumors from normal/overweight patients. A trend toward improved ATP production was also observed in the obese group compared to the normal/overweight group. A trend toward improved ATP production was also observed in the obese group. We did not find significant differences in mitochondrial bioenergetics when patients were stratified by baseline renal function, or tumor histologic grade and stage.

These findings support the hypothesis that obesity induces a distinct metabolic state in ccRCC tumors that may mitigate mitochondrial dysfunction. This adiposity-driven tumor phenotype may confer differential bioenergetic flexibility, influencing disease progression and therapy response. Ongoing mechanistic studies will explore how obesity reshapes the renal tumor microenvironment and whether these metabolic adaptations can be therapeutically leveraged. Understanding these obesity-related vulnerabilities could inform precision treatment strategies for RCC and other obesity-associated cancers.

# Abstract: Performance of a validated 15-gene prognostic signature in localized papillary renal cell carcinoma

**LEAD AUTHOR**

Monda, Steven, University of Michigan

**CO-AUTHORS**

Sophia Abusamra, University of Oxford

Zayne Knuth, University of Michigan

Srinivas Nallandhigal, University of Michigan

Piroz Bahar, University of Michigan

Udit Singhal, University of Michigan

Rohit Mehra, University of Michigan

Hiroshi Kikuchi, University of Michigan

Samuel Kaffenberger, University of Michigan

Khaled Hafez, University of Michigan

Aaron Udager, University of Michigan

Saravana Dhanasekaran, University of Michigan

Marcin Cieslik, University of Michigan

Ganesh Palapattu, University of Michigan

Todd Morgan, University of Michigan

Simpa Salami, University of Michigan

Introduction: Papillary renal cell carcinoma (pRCC) comprises 10-20% of all RCCs. However, progress in developing treatments and diagnostics for pRCC have lagged behind clear cell RCC. Currently, assessing pRCC prognosis relies on clinicopathologic variables alone. Our group has previously developed and validated a 15-gene (15G) prognostic signature for risk stratifying patients with ccRCC (PMID: 38810179 & 40479621). Given that both pRCC and ccRCC arise from the proximal convoluted tubules and share some clinical similarities, we sought to test the performance of our previously validated 15G signature in a pRCC cohort.

Methods: Patients with localized pRCC following nephrectomy and with TNM staging and RNAseq data available (n=245) were identified within The Cancer Genome Atlas (TCGA) for analyses. We calculated 15G scores for each patient utilizing the same methodology as in its original development and validation in ccRCC. We redefined a cut-off of 3.99 for 15G high status in pRCC using the R package surv_cut. To evaluate the association of the 15G signature with disease-free survival (DFS) and disease-specific survival (DSS), we constructed Kaplan-Meier (KM) curves comparing low versus high 15G score.We performed Cox proportional hazards analyses controlling for age, sex, and T-stage using the R package survival to investigate the independent association of categorical and continuous 15G score with DFS.

Results: After a median follow up of 24.5 months, 31 of 245 of patients developed recurrence. As shown in Figure 1A and 1C, patients with high 15G score had shorter DFS and DSS compared to those classified as low risk (p <0.001, p=0.011, respectively). In Cox proportional hazards testing, the 15G high group was independently associated with DFS (hazard ratio [HR], 1.10 [95% CI, 1.02-1.2, p=0.015]) while controlling for age, sex, and T-stage (Figure 1B). A continuous 15G score (+1 point) was also independently associated with worse DFS ([HR] 1.10, [95% CI, 1.02 –1.2], p=0.015) [Figure 1D]. And the inclusion of 15G score improved C-index from 0.69 to 0.73. Notably, 15G high status was significantly associated with advanced tumor characteristics such as somatic mutations, CpG island methylator phenotype (CIMP) status and higher T-stage (p<0.001, p=0.042, p=0.001, respectively).

Conclusions: Here, we report an independent association of a previously validated 15G score for prognosis in ccRCC with DFS and DSS in a cohort of patients with localized pRCC following nephrectomy. With further validation, this gene-expression signature could potentially risk stratify patients with pRCC into high or low risk of recurrence following nephrectomy, thus informing intensity or duration of monitoring and perhaps in the future, potential need for adjuvant therapy.



# Abstract: Equity in Genomic Integration and Outcomes in DDR-Mutant mRCC

**LEAD AUTHOR**

Shakhnazaryan, Nonna, Helen Diller Family Comprehensive Cancer Center, University of California San Francisco, San Francisco, CA

**CO-AUTHORS**

Natalia Sanchez, UCSF Department of Pathology

Kelly Fitzgerald, Helen Diller Family Comprehensive Cancer Center, University of California San Francisco, San Francisco, CA

Deepika Sirohi, UCSF Department of Pathology

Li Zhang, Helen Diller Family Comprehensive Cancer Center, University of California San Francisco, San Francisco, CA

Steven Seyedin, UCSF Department of Radiation Oncology

Arpita Desai, Helen Diller Family Comprehensive Cancer Center, University of California San Francisco, San Francisco, CA

Background: DNA damage repair (DDR) mutations are prognostic and predictive biomarkers in metastatic renal cell carcinoma (mRCC) with implications for targeted therapy. However, genomic results are inconsistently integrated into treatment planning, and access to biomarker-informed care may be shaped by social determinants. We evaluated treatment patterns, genomic testing timelines, and survival outcomes in DDR-mutant mRCC, focusing on social vulnerability.

Methods: We performed a single-institution retrospective review of 17 patients with mRCC and confirmed pathogenic DDR mutations (ClinVar/pathologist-reviewed) treated between 2014–2025. Data included DDR gene, biallelic vs monoallelic status, treatment regimen, radiographic response (RECIST 1.1), and overall survival (OS, from first systemic therapy to death/last follow-up). ZIP code–linked Social Vulnerability Index (SVI) was categorized as low (0.0–0.25), medium (0.26–0.69), or high (≥0.70). Kaplan–Meier estimates and log-rank tests compared OS across groups.

Results: Median age was 70 years (IQR 63–77); 88.2% were male, and most were White (58.8%), Asian (23.5%), or Hispanic/Latino (17.6%). Common DDR alterations: BRCA2 (23.5%), ATM (11.8%), RAD50 (11.8%), and MUTYH (11.8%). First-line therapy was IO + TKI in 53%, IO alone in 24%, TKI alone in 12%, and other regimens in 12%. Median OS was 29.0 months (95% CI 16.6–41.5). Biallelic mutations had shorter OS than monoallelic (11.1 vs 27.3 months). By SVI, median OS was 23.0 months (low), 23.1 months (medium), and 11.4 months (high; p=0.78). Four patients (23.5%) began systemic therapy before DDR results, 75% of whom lived in medium/high-SVI areas.

Conclusion: In DDR-mutant mRCC, high-SVI patients had shorter survival and more often initiated systemic therapy before genomic results, suggesting potential inequities in timely precision oncology. Integrating social determinants into clinical workflows and prioritizing early genomic testing in vulnerable populations may improve equitable access to biomarker-driven care.



# Abstract: Knowledge Gaps and Shared Decision Making (SDM) Disparities in Kidney Cancer (KC) Across North America (NA): Results from the International Kidney Cancer Coalition (IKCC) Global Patient Survey (GPS)

**LEAD AUTHOR**

Jonasch, Eric, MD Anderson Cancer Center, Houston, Texas, USA

**CO-AUTHORS**

Deborah Maskens, International Kidney Cancer Coalition (IKCC), Netherlands

Stênio de Cássio Zequi, A.C. Camargo Cancer Center, Sao Paulo, Brazil

Christine Collins, Kidney Cancer Canada, Ontario, Canada

Omar de Jesus, Asociación ALE, Mexico City, Mexico

Margaret Hickey, International Kidney Cancer Coalition (IKCC), Netherlands

Robin Martinez, Smart Patients Inc., USA

Michael Jewett, Princess Margaret Cancer Centre and Department of Surgery, University of Toronto, Canada, Department of Urology

SDM among patients, their caregivers, and healthcare providers (HCP) has become the standard for cancer care to improve patient outcomes. The IKCC and its network have conducted a biennial GPS since 2018 to assess patient/caregiver experiences on KC burden, diagnosis, and management, to identify unmet needs and country variances, and guide recommendations and actions to close gaps. We present 2025 GPS data on treatment barriers, knowledge gaps, and SDM disparities across NA compared to global responses.

The 2025 GPS was designed by an IKCC steering committee of patient advocates, medical experts and the Picker Institute, and targeted KC patients and carers. It was cognitively tested, translated into 16 languages, and hosted online. Data were analysed using cross-tabulations.

Between Sept 24 and Nov 15, 2024, 2677 responses were received from patients (n=2049) and carers (n=628) from 46 countries, including Canada (n=266), the US (n=220), and Mexico (n=131).

Globally, 55% of respondents reported at least one barrier to treatment versus US and Canada (44% each), and Mexico (81%), with barriers varying across countries.

Knowledge gaps on disease characteristics, treatments, and prognosis were observed. Mexico had the least involvement in SDM (35%) versus global (55%), US (75%), and Canada (67%). Respondents reported support sources varied across countries.

The IKCC GPS is the only worldwide KC survey measuring the experiences of people affected by KC, with a record number of respondents in 2024. Across NA, many patients/carers lack knowledge about their disease, treatment, and prognosis, which can limit SDM involvement. HCPs need to provide clear information to patients/carers about their disease and treatment options to allow them to participate in SDM.

# Abstract: Socioeconomic Disadvantage is Predicted from AI Image Analysis of CT Imaging in Kidney Cancer Patients

**LEAD AUTHOR**

Knorr, Jacob, Cleveland Clinic

**CO-AUTHORS**

Gabriela Diaz, Cleveland Clinic

Rikhil Seshandri, Cleveland Clinic

Haya Abusafieh, Cleveland Clinic

Rishi Jonnalagadda, Cleveland Clinic

Daniel Jevnikar, Cleveland Clinic

Sahil Patel, Cleveland Clinic

Robert Abouassaly, Cleveland Clinic

Nicholas Heller, Cleveland Clinic

Christopher Weight, Cleveland Clinic

Introduction: Socioeconomic status (SES) has been linked to higher rates of mortality after major urologic surgery; however, SES is difficult to assess at the individual level with current measures assessed at the neighborhood level. Artificial intelligence (AI) provides the ability to appreciate additional clinical information from vast amounts of data to make clinical inferences and predictions. For example, AI powered image analysis has shown that predicted “biologic age” is a stronger predictor of survival and chemotherapy tolerance than chronological age in kidney cancer patients. In this study, our objective was to apply AI image analysis to predict Area Deprivation Index (ADI) score and to evaluate the impact of predicted ADI outcomes after nephrectomy.

Methods: Retrospectively, patients who underwent nephrectomy and had preoperative contrast-enhanced abdominal CT imaging available from 10/2009 to 7/2024. ADI, a validated metric for SES, was obtained from the Neighborhood Atlas. A ResNet-50 neural network was fine-tuned to predict ADI from CT image inputs. 5-fold cross validation was used to obtain predictions for all patients. Predicted ADI was compared to actual ADI with linear regression.

Results: A total of 1349 nephrectomy patients had available imaging and ADI. Predicted ADI was correlated with actual ADI in Figure 1.(r = 0.18, p = 1.79x10-11, y = 0.0552 * x + 0.4661). Predicted ADI was not associated with oncologic or survival outcomes following nephrectomy.

Conclusions: On preliminary analysis of a computer-vision based AI model, we demonstrated the ability to predict ADI from CT images in kidney cancer patients, suggesting socioeconomic status may be embedded within medical imaging data. Further refinement of the model is needed. A robust model for SES prediction may provide a better and more personalized estimation of SES exposures and may offer critical insights into how these exposures impact clinical outcomes in patients afflicted by oncologic diseases.



# Abstract: Kidney Cancer Trends, Risk Factors, and Interventions in American Indian and Alaska Native Populations

**LEAD AUTHOR**

La Rosa, Salvatore, Kidney Cancer Association, Houston, TX

**CO-AUTHORS**

Pavlos Msaouel, The University of Texas MD Anderson Cancer Center, Houston, TX

AJ Sedgewick, Tempus AI, Inc., Chicago, IL

Nathan Maulding, Tempus AI, Inc., Chicago, IL

Alejandro Recio Boiles, The University of Arizona Cancer Center, Tucson, AZ

Ken Batai, Roswell Park Comprehensive Cancer Center, Buffalo, NY

American Indian and Alaska Native (AI/AN) populations experience disproportionately high kidney cancer incidence and mortality rates compared to other U.S. groups. Literature was reviewed to explore the factors contributing to the unequally higher kidney cancer burden in AI/AN communities and to develop recommendations to reduce these disparities. The incidence of kidney cancer has been rising over the past few decades, and this increase has been especially steep among AI/AN individuals. Death rates in AI/AN populations are roughly twice those of the non-Hispanic White population. The elevated kidney cancer burden in AI/AN may be driven by both clinical risk factors (obesity, diabetes, hypertension, chronic kidney disease, smoking, and environmental factors) and structural determinants of health which can critically shape these disparities. Chronic health conditions and smoking are linked to poverty and education level. AI/AN communities have high uninsured rates, and the Indian Health Services is chronically underfunded. Systemic inequalities limit AI/AN patients and community members’ access to chronic disease management, smoking cessation programs, primary and specialty care for early detection, and ultimately, treatment. AI/AN patients may have mistrust or other cultural barriers to engaging with the healthcare system and providers, while implicit bias in healthcare providers may lead to undertreatment, key interventions and tailored programs aimed at reducing kidney cancer incidence and mortality are needed. Here we will highlight some current interventions, including access to disease management and smoking cessation programs; facilitating health care access and quality; adopting patient navigation and culturally competent education; and developing strategies for early detection, such as screening in high-risk populations. In partnership with AI/AN communities, a combination of prevention, early detection, and healthcare system improvements is needed to close the kidney cancer gap between AI/AN and other populations.



# Abstract: External Validation of AI Age Discrepancy as a Measure of Frailty in Kidney Tumor Patients

**LEAD AUTHOR**

Seshadri, Rikhil, Cleveland Clinic

**CO-AUTHORS**

Haya Abusafieh, Cleveland Clinic

Rishi Jonnalagadda, Cleveland Clinic

Sahil Patel, Cleveland Clinic

Daniel Jevnikar, Cleveland Clinic

Salim Younis, Cleveland Clinic

Abdulrahman Al Bayati, Cleveland Clinic

Nicolas Soputro, Cleveland Clinic

Jacob Knorr, Cleveland Clinic

Betty Wang, Cleveland Clinic

Gagan Fervaha, Cleveland Clinic

Michal Ozery Flato, IBM Research

Michal Rosen Zvi, IBM Research

Robert Abouassaly, Cleveland Clinic

Erick Remer, Cleveland Clinic

Nicholas Heller, Cleveland Clinic

Christopher Weight, Cleveland Clinic

Recently-published work introduces AI Age Discrepancy, a novel parameter for quantifying frailty by quantifying the discrepancy between a patient’s true age, and the age that an AI system predicts for them based on their abdominal CT scan. It was found to predict shorter overall survival (OS) and longer length of stay (LOS), independent of established factors. This study replicates those findings in a larger cohort from a different health system, along with an external validation. This retrospective study included 1800 patients treated at a single large health system (internal cohort) and 590 patients from an external validation dataset. All underwent contrast-enhanced CT imaging and underwent partial or radical nephrectomy for suspected renal malignancy. Patients under 18 were excluded. A ResNet-50 neural network was fine-tuned to predict age from CT images. Using 5-fold cross-validation, age predictions were obtained and averaged for ensemble predictions. Multivariate Cox proportional-hazards regression was used to evaluate AI Age Discrepancy as a predictor of LOS and OS, adjusting for established factors as model covariates. Model-predicted ages were highly correlated with true age for both the internal (r=0.75, p=1.86e-321) and external (r=0.71, p=2.81e-91) cohorts. In the internal cohort, AI Age discrepancy significantly predicted OS (HR=1.021, p=0.013), but not LOS. In the external validation cohort, it was a significant predictor of both OS (HR=1.073, p=0.023) and LOS (HR=0.985, p=0.015). AI Age Discrepancy is a significant and generalizable predictor of overall survival in kidney cancer patients undergoing nephrectomy. Its predictive value for LOS may vary by institution. These findings support its integration into preoperative risk assessment to better individualize patient management and inform shared decision-making.



# Abstract: Pathologic T3 RCC Prediction Using the SHARP (Staging of High-risk And nonmetastatic Renal cell carcinoma Preoperatively) Model: A Tool for Preoperative Staging and Neoadjuvant Trial Enrollment

**LEAD AUTHOR**

Lotan, Paz, The University of Wisconsin, School of Medicine and Public Health, Madison, WI, USA

**CO-AUTHORS**

Daniel D. Shapiro, The University of Wisconsin, School of Medicine and Public Health, Madison, WI, USA

Andrew L. Wentland, The University of Wisconsin, School of Medicine and Public Health, Madison, WI, USA

Matthew R. Smith, The University of Wisconsin, School of Medicine and Public Health, Madison, WI, USA

Glenn O. Allen, The University of Wisconsin, School of Medicine and Public Health, Madison, WI, USA

David F. Jarrard, The University of Wisconsin, School of Medicine and Public Health, Madison, WI, USA

Kye Nichols, The University of Wisconsin, School of Medicine and Public Health, Madison, WI, USA

Danny Roadman, The University of Wisconsin, School of Medicine and Public Health, Madison, WI, USA

Michael C. Risk, The University of Wisconsin, School of Medicine and Public Health, Madison, WI, USA

Kyle A. Richards, The University of Wisconsin, School of Medicine and Public Health, Madison, WI, USA

Elizabeth Koehne, The University of Wisconsin, School of Medicine and Public Health, Madison, WI, USA

E. Jason Abel, The University of Wisconsin, School of Medicine and Public Health, Madison, WI, USA

Background: Given the success of modern systemic therapies for metastatic renal cell carcinoma, recent clinical trials are increasingly investigating neoadjuvant therapy for high-risk non-metastatic tumors >7cm. Although pathologic stage T3 (pT3) is associated with a significantly higher risk of metastatic progression compared to lower-stage tumors, few studies have evaluated the ability to accurately identify pT3 stage tumors >7cm using preoperative imaging. This study aimed to develop a diagnostic model to identify pT3 in non-metastatic RCC >7cm using radiologic features on preoperative computed tomography (CT) imaging.

Methods: Data were analyzed for consecutive patients with non-metastatic RCC >7cm treated with nephrectomy from 2000 to 2024 with available preoperative CT scans obtained within 3 months before surgery. Three readers independently evaluated the preoperative CT for radiographic features (Figure 1A) including perinephric stranding, irregular tumor-fat interface, peritumor neovascularity, venous tumor thrombus, and increased peritumoral fat density (≥15HU). A multivariable model was developed, incorporating significant radiographic features associated with pT3. The model's predictive accuracy and clinical utility were evaluated.

Results: A total of 326 patients were included. The median age at surgery was 62 years. The median tumor size was 9.1cm, and 192 patients (59%) had pT3 stage. Four out of five radiographic features were associated with pT3 stage and included in the final multivariable model with perinephric stranding excluded (Figure 1B). The model demonstrated predictive accuracy with an AUC of 0.84 (95% CI 0.80-0.88). Decision curve analysis indicated clinical utility across a wide range of threshold probabilities (Figures 1C and 1D).

Conclusions: The SHARP model predicts pT3 RCC in tumors >7cm using preoperative imaging and offers increased clinical utility compared to size stratification alone to identify patients at higher risk for recurrence events. With validation, the SHARP model could be used to improve clinical staging and optimize patient stratification for neoadjuvant clinical trial enrollment.



# Abstract: Artificial Intelligence to Predict Risk of Chronic Kidney Disease Progression After Nephrectomy Based on Preoperative Cross-Sectional Imaging

**LEAD AUTHOR**

Patel, Sahil, Case Western Reserve University

**CO-AUTHORS**

Rikhil Seshadri, Cleveland Clinic

Haya Abusafieh, Cleveland Clinic

Rishi Jonnalagadda, Cleveland Clinic

Daniel Jevnikar, Cleveland Clinic

Jessica Yoon, Cleveland Clinic

Jacob Knorr, Cleveland Clinic

Gagan Fervaha, Cleveland Clinic

Michal Ozery Flato, IBM Research

Michal Rosen Zvi, IBM Research

Robert Abouassaly, Cleveland Clinic

Erick Remer, Cleveland Clinic

Nicholas Heller, Cleveland Clinic

Christopher Weight, Cleveland Clinic

Chronic kidney disease (CKD) is a common and serious complication following nephrectomy for renal tumors. Preoperative estimation of renal function is critical for surgical planning, risk counseling, and long-term management. Existing models use parenchymal volume estimation or multivariable regression with baseline estimated glomerular filtration rate (eGFR), age, comorbidities, and surgical approach. AUA guidelines advise caution when patients’ eGFRs are less than 45 mL/min/1.73m2 or when eGFR is expected to decrease significantly after intervention. This study evaluates a computer vision model for predicting eGFR decline after nephrectomy, supporting risk stratification and shared decision-making. This retrospective study included 1,682 patients who underwent nephrectomies for renal tumors at a single large health system. A ResNet-50 convolutional neural network was trained to predict 1-year postoperative CKD stage based on preoperative CT imaging. A subanalysis of 1,428 patients with preoperative eGFR >= 45 was conducted. A multivariate regression model was developed using the predicted 1-year postoperative CKD stage, preoperative eGFR, age, and surgical type (radical versus partial nephrectomy) to identify patients who were at risk of progressing to an eGFR < 45 at 1-year post-nephrectomy. The model’s AUC was compared to those of existing models using parenchymal volume analysis and a clinical risk calculator. AUC differences were assessed with DeLong’s test. Median preoperative eGFR was 59, and median age was 65. Radical nephrectomy was performed in 37.7% and 62.3% had nephron-sparing surgery. The multivariable regression model had an AUC of 0.832 in predicting eGFR <45. The clinical calculator had an AUC of 0.817 (p=0.206), and the volume estimation model had an AUC of 0.749 (p=0.001). This AI model accurately predicts CKD progression from imaging and labs, potentially improving risk assessment, counseling, and surgical planning. Prospective, multi-center validation is needed before clinical integration.



# Abstract: External Validation of an AI-Based Automatic Renal Nephrometry Scoring System

**LEAD AUTHOR**

Jonnalagadda, Rishi,Cleveland Clinic

**CO-AUTHORS**

Daniel Jevnikar, Cleveland Clinic

Sahil Patel, Case Western Reserve University

Rikhil Seshadri, Cleveland Clinic

Haya Abusafieh, Cleveland Clinic

Jacob Knorr, Cleveland Clinic

Betty Wang, Cleveland Clinic

Gagan Fervaha, Cleveland Clinic

Michal Ozery Flato, IBM Research

Michal Rosen Zvi, Cleveland Clinic

Robert Abouassaly, Cleveland Clinic

Erick Remer, Cleveland Clinic

Nicholas Heller, Cleveland Clinic

Christopher Weight, Cleveland Clinic

The R.E.N.A.L. nephrometry score standardizes renal tumor assessment for surgical planning and prognostication. However, manual scoring is limited by interobserver variability and inefficiency. Continuous AI-derived scores have been shown to improve reproducibility and predictive accuracy for outcomes such as malignancy and surgical approach compared to the conventional ordinal categories assigned by human raters. This study aimed to validate an AI-based R.E.N.A.L. scoring system in a large, independent cohort from a single healthcare system. A ResNet-50 neural network was previously trained to predict numeric R.E.N.A.L. score components using preoperative CT images and expert segmentation masks from 599 patients in a single-institution cohort. For external validation, the model was applied to preoperative CT scans from 1,806 patients at another large healthcare system. ROC analysis assessed the association between automated nephrometry scores and surgical or pathological outcomes, including radical or partial nephrectomy, open surgery, pT stage 3+, and ISUP grade 3+. Human-determined R.E.N.A.L. scores were available for 193 patients, enabling direct comparison. AI-predicted composite R.E.N.A.L. scores demonstrated high correlation with human scoring in this subset. The automated scores also showed predictive potential for key clinical outcomes: radical or partial nephrectomy (AUC = 0.78), high pathological stage (T3+) (AUC = 0.72), High ISUP Grade (G3+) (AUC = 0.64), and open surgery (AUC = 0.59). By providing continuous, reproducible outputs, the AI model offers an alternative to manual scoring that may reduce variability and enhance prognostic performance. These findings support the integration of AI-generated nephrometry scores into radiology workflows, enabling faster, more standardized assessments that can inform surgical decision-making.

# Abstract: Artificial Intelligence for Predicting Perioperative Outcomes From Preoperative Imaging in Kidney Tumor Patients

**LEAD AUTHOR**

Seshadri, Rikhil, Cleveland Clinic

**CO-AUTHORS**

Haya Abusafieh, Cleveland Clinic

Sahil Patel, Case Western Reserve University

Daniel Jevnikar, Cleveland Clinic

Rishi Jonnalagadda, Cleveland Clinic

Salim Younis, Cleveland Clinic

Abdulrahman Al Bayati, Cleveland Clinic

Nicolas Soputro, Cleveland Clinic

Jacob Knorr, Cleveland Clinic

Betty Wang, Cleveland Clinic

Gagan Fervaha, Cleveland Clinic

Michal Ozery Flato, IBM Research

Michal Rosen Zvi, IBM Research

Robert Abouassaly, Cleveland Clinic

Erick Remer, Cleveland Clinic

Nicholas Heller, Cleveland Clinic

Christopher Weight, Cleveland Clinic

Partial nephrectomy (PN) is the preferred surgical approach for localized kidney tumors when feasible, preserving renal function but carrying a slightly higher risk of postoperative complications and longer operative times compared to radical nephrectomy (RN). Additional perioperative factors, including prolonged warm ischemia time and significant intraoperative blood loss, can contribute to long-term renal impairment and worse outcomes. Accurately predicting these risks is critical for selecting the optimal surgical strategy. Nephrometry scores such as RENAL quantify surgical complexity but are limited by human-defined heuristics and modest correlation with perioperative outcomes. We developed an AI-based image analysis approach to predict the likelihood of PN vs RN, as well as expected ischemia time and estimated blood loss, from preoperative CT imaging. We retrospectively analyzed 1,641 nephrectomy patients from a single large health system with available preoperative contrast-enhanced abdominal CT scans and perioperative data (10/2009-8/2024). A ResNet-50 model was fine-tuned to predict RN vs. PN using 5-fold cross-validation. Subanalysis was performed on tumors 3-7cm to evaluate performance in cases with greater diagnostic uncertainty. Predictions were compared with AI-estimated RENAL scores and tumor size using AUC-ROC and DeLong’s test. Regression models assessed correlations with ischemia time and blood loss, with comparisons using Pearson correlation and Steiger’s test. The model’s AUC was 0.88 for predicting RN, outperforming tumor size (AUC 0.86, p=0.008) and RENAL score (AUC 0.80, p=3.2e-12). In the 3-7cm cohort, the AUC was 0.78, also exceeding tumor size (0.73, p=0.001) and RENAL (0.72, p = 4.0e-4). AI-based predictions also correlate more strongly than RENAL scores with estimated blood loss and ischemia time. These findings demonstrate the potential of AI-driven imaging analysis to improve surgical planning, and risk stratification in kidney cancer surgery, with future validation needed for clinical adoption.

# Abstract: Predicting Clear Cell Subtype for Kidney Tumors From Cross-Sectional Imaging Using Artificial Intelligence

**LEAD AUTHOR**

Abusafieh, Haya, Cleveland Clinic

**CO-AUTHORS**

Rikhil Seshadri, Cleveland Clinic

Sahil Patel, Case Western Reserve University

Rishi Jonnalagadda, Cleveland Clinic

Daniel Jevnikar, Cleveland Clinic

Salim Younis, Cleveland Clinic

Abdulrahman Al Bayati, Cleveland Clinic

Nicolas Soputro, Cleveland Clinic

Jacob Knorr, Cleveland Clinic

Gagan Fervaha, Cleveland Clinic

Michal Ozery Flato, IBM Research

Michal Rosen Zvi, IBM Research

Robert Abouassaly, Cleveland Clinic

Erick Remer, Cleveland Clinic

Nicholas Heller, Cleveland Clinic

Christopher Weight, Cleveland Clinic

Clear cell renal cell carcinoma (ccRCC) is the most common subtype of kidney cancer in adults. The standard of care for localized RCC is partial or radical nephrectomy or active surveillance for small tumors. Histologic diagnosis can assist shared decision-making, but is typically unavailable prior to renal mass biopsy, which has limitations due to tumor heterogeneity. This study explores whether histopathologic diagnoses can be predicted based on radiomic data. We present the application of artificial intelligence (AI) to predict the clear cell subtype directly from preoperative imaging. We retrospectively reviewed patients who underwent nephrectomy with available preoperative contrast-enhanced abdominal CT imaging from 10/2009 to 7/2024 at a single large health system. A ResNet-50 architecture was fine-tuned to predict ccRCC, using a 5-fold cross-validation. Additional models were trained on tumors sized 3-7cm. The ensemble of 5 models was validated on an independent external dataset. AUC comparisons between the AI model and a tumor size-based prediction model were evaluated using DeLong’s test. Among 1642 nephrectomy patients with imaging and subtype classifications, 822 had tumors 3-7 cm. In the whole cohort, the model achieved an AUC of 0.71, significantly outperforming the tumor size AUC of 0.54 (p=6.6e-21). For the 3-7cm subset, the model achieved an AUC of 0.81, outperforming tumor size (AUC of 0.49; p=2.1e-26). On the external validation cohort, the model also outperformed tumor size in the 3-7cm subset (AUCs of 0.59 vs 0.44; p=0.009), but not significantly in the full external cohort. A computer-vision AI model can predict ccRCC from CT imaging more accurately than tumor size, offering a potential non-invasive alternative to biopsy for presurgical decision-making in kidney cancer. This may support treatment planning in cases where knowing the tumor subtype could influence the management approach.



# Abstract: Beyond Clear Cell: Rethinking Postoperative Surveillance for Non-clear cell RCC

**LEAD AUTHOR**

Das, Arighno, Department of Urology, The University of Texas MD Anderson Cancer Center, Houston, Texas

**CO-AUTHORS**

Elizabeth Ellis, Department of Urology, The University of Texas MD Anderson Cancer Center, Houston, Texas

Pheroze Tamboli, Department of Pathology, The University of Texas MD Anderson Cancer Center, Houston, Texas

Priya Rao, Department of Pathology, The University of Texas MD Anderson Cancer Center, Houston, Texas

Surena Matin, Department of Urology, The University of Texas MD Anderson Cancer Center, Houston, Texas

Jose Karam, Department of Urology, The University of Texas MD Anderson Cancer Center, Houston, Texas

Brittney Cotta, Department of Urology, The University of Texas MD Anderson Cancer Center, Houston, Texas

INTRODUCTION: Current American Urologic Association (AUA) and National Comprehensive Cancer Network (NCCN) guidelines do not differentiate between clear-cell RCC and non-clear cell RCC (nccRCC), while European Urologic Association (EAU) does. Long-term recurrence data for nccRCC remains limited. We analyzed recurrence patterns after surgery for nccRCC and compared the prognostic performance of the guidelines.

METHODS/MATERIALS: We identified 611 adults treated surgically for nonmetastatic, non-hereditary nccRCC (2003-2013). Eligible histologies included papillary, chromophobe, translocation, mucinous tubular/spindle cell carcinoma, and unclassified RCC. Clinicopathologic factors, recurrence-free survival (RFS) and first-recurrence were recorded. Patients were stratified by guideline risk groups; c-index and integrated Brier score (IBS) assessed model performance.

RESULTS: Papillary RCC was the most common (364/611, 60%). Median follow-up was 100 months (IQR 36, 167). Fifty-eight patients (9.5%) recurred at a median of 13.5 months (IQR 6, 45). Recurrences were mainly intra-abdominal (38/58, 65%) particularly in retroperitoneal nodes (24/58, 41%); thoracic recurrences were seen in 22/58 (38%). One- and five-year RFS were 95% (95% CI 93–97) and 92% (89–94). After stage adjustment, chRCC had lowest risk of recurrence (HR 0.24, 95% CI 0.11–0.55). Most patients were AUA/EAU low risk (266/611, 44%) or NCCN Stage 1 (414/611, 68%). Survival was significantly different between risk groups across guidelines (Figure 1). AUA risk strata offered marginally superior discrimination (C-index) and calibration (IBS). Among AUA/EAU low-risk patients, only one recurrence occurred (0.3%). In intermediate-risk groups, 5.4% (AUA) and 3.3% (EAU) recurred, with most events after five years (77%).

CONCLUSIONS: Recurrence after nccRCC surgery is uncommon but more often intra-abdominal than thoracic. Late recurrence events are frequent in intermediate-risk patients. A risk-adapted approach—shortened surveillance for low-risk and ≥10-year follow-up for higher-risk groups—could reduce imaging without sacrificing detection. All three systems stratify risk; AUA provides the best discrimination and calibration.



# Abstract: Single Institution Analysis of Outcomes in Chromophobe Renal Cell Carcinoma, Oncocytoma, and Oncocytic Neoplasm of Low Malignant Potential

**LEAD AUTHOR**

Knorr, Jacob, Cleveland Clinic

**CO-AUTHORS**

Annaliese Ionson, Cleveland Clinic

Vishu Chadrasekhar, Cleveland Clinic

Jason Scovell, Cleveland Clinic

Michael Gross, Cleveland Clinic

Gabriela Diaz, Cleveland Clinic

Nicholas Heller, Cleveland Clinic

Reza Alaghehbandan, Cleveland Clinic

Christopher Weight, Cleveland Clinic

Venkatesh Krishnamurthi, Cleveland Clinic

INTRODUCTION

Eosinophilic or “pink” renal tumors typically follow an indolent course with low recurrence and metastasis rates. However, there remains uncertainty risk stratification of tumors across this spectrum, with potential oncologic differences among chromophobe renal cell carcinoma (chRCC), oncocytoma, and oncocytic neoplasm of low malignant potential (ORNLMP). Furthermore, there are limited studies assessing comparative outcomes in these respective pathologies following partial or radical nephrectomy. The objective of this study was to evaluate clinical outcomes among these eosinophilic renal tumors and identify factors associated with adverse oncologic outcomes in a pathology-confirmed cohort.

METHODS

We conducted a retrospective review of our institutional database to examine clinical outcomes in patients with chRCC, oncocytoma, and ORNLMP. Cases were re-reviewed by pathology to confirm histopathologic diagnosis. Chromophobe tumors were stratified by stage for analysis. Outcomes of interest included disease metastasis and patient survival.

RESULTS

We identified 867 patients with chRCC (n=437, 50.4%), oncocytoma (n=345, 39.8%), or ORNLMP (n=85, 9.8%) who received either partial or radical nephrectomy from 1996 to 2021, Median follow up was 80 months. Rates of metastasis for chRCC (21/416, 4.8%) were significantly higher than oncocytoma (0/345, 0%), and ORNLMP (0/85, 0%) (p=<0.001). Metastasis free survival and overall survival were significantly worse for T3a chRCC compared to oncocytoma, ORNLMP, and T2b chRCC (Figure 1.).

CONCLUSIONS

The findings of our large single center study support previous studies which demonstrate that both oncocytoma and ORNLMP do not recur with prolonged follow-up. As expected, patients with higher stage chRCC have poorer oncologic outcomes compared to lower stage chRCC. Conversely, lower stage chRCC behave similar to ORNLMP and oncocytoma. Further studies are warranted to explore risk factors for metastasis in high stage chRCC and compare outcomes with other forms of renal cell carcinoma.



# Abstract: Quality of life (QOL), physician-selected reasons, and starting doses for immunooncology (IO)-based systemic therapy (ST) in the ODYSSEY prospective observational study

**LEAD AUTHOR**

Harrison, Michael R., Duke Cancer Institute Center for Prostate and Urologic Cancers

**CO-AUTHORS**

Yasser Ged, The Sidney Kimmel Comprehensive Cancer Center at Johns Hopkins

Benjamin L. Maughan, University of Utah Huntsman Cancer Institute

Elizabeth Wulff, University of Kansas Health System

Nrupen Bhavsar, Duke University School of Medicine, Department of Surgery

Leonard Appleman, UPMC Hillman Cancer Center

Yousef Zakharia, Mayo Clinic Comprehensive Cancer Center

Brian Costello, Mayo Clinic Comprehensive Cancer Center

Deepak Kilari, Froedtert & the Medical College of Wisconsin Clinical Cancer Center

Sorab Gupta, Geisinger Health System

Melyssa Bratton, Ochsner Health Tom and Gayle Benson Cancer Center

Fernando Barbosa, University of Iowa

Paul Monk, The Ohio State University Comprehensive Cancer Center

Ulka Vaishampayan, Rogel Cancer Center, University of Michigan

Tracy Rose, UNC Lineberger Comprehensive Cancer Center

Sarah Jabusch, Duke Clinical Research Institute

Kimberly T. Ward, Duke Clinical Research Institute

Yuan Wu, Duke Department of Biostatistics & Bioinformatics

Courtney Page, Duke Department of Biostatistics & Bioinformatics

Tian Zhang, UT Southwestern Simmons Comprehensive Cancer Center

Daniel J. George, Duke Cancer Institute Center for Prostate and Urologic Cancers

Background: Cross-trial comparisons of first-line IO-based regimens for metastatic renal cell carcinoma (mRCC) are limited by lack of standardization and reporting of patient-reported outcomes (PROs). Real world data may fill an important knowledge gap.

Methods: ODYSSEY is a multi-site, prospective study of pts with mRCC. Pts must have mRCC (any histology) and no prior ST; those treated for cancer except mRCC are excluded. The primary objective is to determine patterns of change in QOL and symptom burden. Minimally important differences are 3 points for FKSI-19, 1 point for FKSI-DRS and 7 points for FACT-G.

Results: As of 7/1/25, 351 pts were managed with ST, including 257 IO-based combinations: 34 cabozantinib + nivolumab (C+N), 47 lenvatinib + pembrolizumab (L+P), 39 axitinib + pembrolizumab (A+P), and 137 ipilimumab + nivolumab (I+N). Overall median follow-up is 11.8 months (IQR 4.5, 20.9).

Age, sex and race were similar across regimens; prior nephrectomy (50%) was lower than trials. C+N and L+P pts had less clear cell (56 and 67%) versus A+P and I+N pts (94 and 82%). A+P pts had more favorable IMDC risk (and less poor risk).

Baseline PROs, physician-reported weighted reasons for treatment selection, and starting doses are shown in the Table.

Conclusions: Pts on ODYSSEY had lower baseline QOL compared with those on the same regimen in corresponding pivotal trials. The top physician reason for picking an IO-TKI regimen was delaying progression compared to prolonged survival for I+N. Variability in starting TKIs at the FDA-approved starting dose was observed. Since TKI-IO outcomes are linked to dose intensity, this suggests a disconnect between what physicians say (reason) and actually do (dose). Longer follow-up is needed to determine the ramifications of these choices.



# Abstract: Shifting Treatment Patterns in Chromophobe Renal Cell Carcinoma: A U.S. Real-World Data Analysis

**LEAD AUTHOR**

Zugman, Miguel, City of Hope Comprehensive Cancer Center, Duarte, CA USA

**CO-AUTHORS**

Jennifer R. Rider, ConcertAI LLC, Cambridge, MA USA

Nazli Dizman, The University of Texas MD Anderson Cancer Center, Houston, TX USA

Salvador Jaime-Casas, City of Hope Comprehensive Cancer Center, Duarte, CA USA

Daniela V. Castro, City of Hope Comprehensive Cancer Center, Duarte, CA USA

Benjamin Mercier, City of Hope Comprehensive Cancer Center, Duarte, CA USA

Nicholas J. Salgia, Department of Immunology, Roswell Park Comprehensive Cancer Center, Buffalo, NY, USA

Koral Shah, City of Hope Comprehensive Cancer Center, Duarte, CA USA

Vitor Abreu de Goes, City of Hope Comprehensive Cancer Center, Duarte, CA USA

Regina Barragan-Carrillo, City of Hope Comprehensive Cancer Center, Duarte, CA USA

Diwyanshu Sahu, ConcertAI LLC, Bengaluru, Karnataka, India

Prithviraj Vikramsinh Mandora, ConcertAI LLC, Bengaluru, Karnataka, India

JoAnn Hsu, City of Hope Comprehensive Cancer Center, Duarte, CA USA

Wesley Yip, City of Hope Comprehensive Cancer Center, Duarte, CA USA

Charles B. Nguyen, City of Hope Comprehensive Cancer Center, Duarte, CA USA

Alex Chehrazi-Raffle, City of Hope Comprehensive Cancer Center, Duarte, CA USA

Robert Miller, ConcertAI LLC, Cambridge, MA USA

Thomas Powles, Department of Genitourinary Oncology, Barts Health NHS Trust Saint Bartholomew's Hospital, London, UK;

Toni Choueiri, Lank Center for Genitourinary Oncology, Dana-Farber Cancer Institute and Harvard Medical School, Boston, MA, USA

Sumanta K. Pal, City of Hope Comprehensive Cancer Center, Duarte, CA USA

Background: Chromophobe renal cell carcinoma (chRCC) is the second most common variant histology RCC. There are no dedicated prospective studies in this subtype, and evidence supporting contemporary first-line therapies is limited to mixed-histology, single-arm trials. We leveraged real-world data to characterize evolving treatment patterns and associated outcomes in chRCC.

Methods: Patients with chRCC were retrospectively identified from the ConcertAI Patient360TM RCC Dataset, a US-based de-identified real-world dataset derived from electronic medical records, linked claims, and mortality data. Histology was defined by ICD-O-3 codes (8270/3, 8317/3) and NCI System code C40344. Among patients with metastatic disease treated with first-line systemic therapy, regimens were categorized as tyrosine kinase inhibitors (TKIs), immune checkpoint inhibitors (IO, monotherapy or IO/IO), MET inhibitors (METi), mTOR inhibitors (mTORi), or IO-based combinations (IO+TKI or IO+METi). Outcomes included real-world progression-free survival (rwPFS) and overall survival (rwOS), estimated using Kaplan–Meier methods.

Results: A total of 87 patients with metastatic chRCC were identified. Median age at diagnosis was 62.5 years (IQR 52.3–72.8), and 59% were male. Most were treated in community oncology practices (93.1%) and were White (74.7%), with 8.1% Black and 5.8% Hispanic or Latino. At diagnosis, 25.3% had stage IV disease and 68.9% had ECOG 0–1. Among 51 patients with evaluable first-line treatment data, TKIs were most common (43%), followed by METi (15.6%) and IO-based combinations (15.6%). Use of IO and IO-based regimens increased over time. Median rwPFS and rwOS were 5.45 and 21.5 months, respectively. No consistent differences in survival were observed across treatment groups based on qualitative comparison of Kaplan–Meier curves.

Conclusions: In this national real-world cohort of metastatic chRCC, treatment has shifted toward IO and IO-based combinations, though survival outcomes remained comparable across regimens. Histology-focused prospective trials are needed to guide optimal therapy.

# Abstract: Real world experience with adjuvant pembrolizumab in clear cell renal cell carcinoma (ccRCC)

**LEAD AUTHOR**

Al Abbas, Mousab, Mayo Clinic, AZ

**CO-AUTHORS**

Kainat Warrish, Mayo Clinic, AZ

Maurish Fatima, Mayo Clinic, AZ

Mohammed Dheyaa Marsool, Mayo Clinic, AZ

Haidar Abdul-Muhsin, Mayo Clinic, AZ

Irbaz Bin Riaz, Mayo Clinic, AZ

Yousef Zakharia, Mayo Clinic, AZ

Background:

Keynote 564 has led to Pembrolizumab FDA approval as an adjuvant treatment for high-risk clear cell renal cell carcinoma (ccRCC). However, there is limited data describing real-world experience receiving Adjuvant Pembrolizumab.

Methods:

A database query at Mayo Clinic Comprehensive Cancer Center identified 160 ccRCC patients who received pembrolizumab following nephrectomy between 2021 and 2025. Of those, 88 patients had pembrolizumab in accordance to the KEYNOTE-564 trial and were included in the analysis. The remaining 72 patients were excluded due to either receiving pembrolizumab in combination with other therapies for in metastatic setting (68 patients) or not receiving the drug at all (4 patients).

Results:

Of the 88 patients, 70% were male and the median age was 66 years. The median follow-up period was 12 months (range, 1–38). The median interval between nephrectomy and pembrolizumab initiation was 54 days (range, 19-161). Eighteen patients remain on treatment, with a median of 7 cycles received (range, 1–15). Among the remaining 70, only 31 (44.2%) completed the scheduled 17 cycles. The other 39 patients discontinued treatment, most commonly due to adverse events (31 patients), followed by disease recurrence (8 patients). The 31 patients who discontinued the drug due to adverse events received a median of 8 cycles (range, 1 to 16).

A total of 19 disease recurrences were observed, with 13 occurred early during the first year of drug initiation. These patients received a median of 6 cycles (range, 1–13) before switching regimens. Among those with early recurrence, 9 had grade 4 tumors with either sarcomatoid or rhabdoid features. The lungs were the most common site of recurrence.

The estimated percentages of patients who remained alive and recurrence-free were 81.9% (95% CI, 73.3%–91.5%) at 12 months and 69.2% (95% CI, 56.7%–84.5%) at 24 months, based on Kaplan–Meier analysis.

Conclusion:

In this real-world cohort, less than half of patients completed the planned adjuvant pembrolizumab therapy. Notably, most of disease recurrences occurred within the first year of therapy. Our estimates of DFS should be interpreted with caution due to the relatively short median follow-up duration.



# Abstract: Real-world Study of Treatment Patterns in Heavily Pretreated Metastatic Renal Cell Carcinoma (mRCC) Patients

**LEAD AUTHOR**

Shah, Neil, Memorial Sloan Kettering Cancer Center, New York, NY

**CO-AUTHORS**

Sneha Sura, Ontada, Boston, MA, USA

Reshma Shinde, Merck & Co., Inc., Rahway, NJ, USA

Wei Dai, Ontada, Boston, MA, USA

Rodolfo Perini, Merck & Co., Inc., Rahway, NJ, USA

Donna Vickery, Merck & Co., Inc., Rahway, NJ, USA

Robert Motzer, Memorial Sloan Kettering Cancer Center, New York, NY

Background: The treatment landscape for mRCC has evolved with the introduction of immune-oncology (IO) therapies and broader use of tyrosine kinase inhibitors (TKIs). However, treatment decisions for heavily pretreated patients remain complex. This study evaluated real-world treatment patterns and clinical outcomes in patients who received one IO and two TKI-based therapies.

Methods: We conducted a retrospective observational study using structured data from the iKnowMed electronic health record database within The US Oncology Network. Patients with mRCC who initiated a subsequent line of therapy (LOT; index date) after one IO and two TKI-based regimens in combination/sequence between 01/01/2018 and 09/30/2023 were included in the cohort and followed through 03/31/2024. We performed descriptive analyses to assess patient characteristics and treatment patterns. We estimated real-world time on treatment (rwToT), real-world time to next treatment (rwTTNT), and overall survival (OS) using Kaplan-Meier methods.

Results: A total of 293 patients were included (median age: 66 years; 74.7% male; 55.9% ECOG 0–1; 86.3% clear cell histology; 80.8% intermediate/poor IMDC risk). In our cohort, 27.6% initiated index treatment at LOT3, 62.1% at LOT4, and 10.2% at LOT5+. Top 2 index therapy classes were TKI monotherapy (42.3%), with cabozantinib (37.1%) and axitinib (18.5%) most frequently used therapies, followed by TKI + mTORs (19.8%) with lenvatinib + everolimus being the treatment of choice. Overall, median (95% CI) rwToT, rwTTNT, and OS from index date were 3.0 (2.4–3.5), 6.2 (5.4–7.9), and 15.1 (12.5–19.3) months, respectively.

Conclusions: Despite prior exposure to at least two TKIs and one IO, TKI-based therapies were the predominant treatment strategy. This underscores an urgent need for novel anticancer agents with distinct mechanisms of action and more favorable toxicity profiles to optimize outcomes for mRCC patients.



# Abstract: Using Large Language Models To Automatically Extract Structured Data From Radiology, Operative, and Pathology Reports for Kidney Tumor Patients

**LEAD AUTHOR**

Jevnikar, Daniel, Cleveland Clinic

**CO-AUTHORS**

Haya Abusafieh, Cleveland Clinic

Sahil Patel, Case Western Reserve University

Rikhil Seshadri, Cleveland Clinic

Rishi Jonnalagadda, Cleveland Clinic

Jessica Yoon, Case Western Reserve University

Gabriela Diaz, Cleveland Clinic

Salim Younis, Cleveland Clinic

Abdulrahman Al Bayati, Cleveland Clinic

Nicolas Soputro, Cleveland Clinic

Jacob Knorr, Cleveland Clinic

Betty Wang, Cleveland Clinic

Gagan Fervaha, Cleveland Clinic

Michal Ozery Flato, IBM Research

Michal Rosen Zvi, IBM Research

Nicholas Heller, Cleveland Clinic

Christopher Weight, Cleveland Clinic

Robert Abouassaly, Cleveland Clinic

The construction of large-scale clinical databases using Electronic Medical Records (EMRs) poses a significant challenge, particularly when extracting information from unstructured documents such as operative, radiology, and surgical pathology notes. Manual data abstraction is time-consuming, error-prone, and inconsistent, with reported data extraction errors ranging between 8% and 42%. Large language models (LLMs) show promise in understanding free-text and extracting relevant clinical variables. This study evaluates the performance of Meta AI’s LLaMA 3.0-8B model in extracting kidney cancer variables from clinical notes across a large, multi-institutional nephrectomy cohort. We applied the model to 8,366 patients who underwent a nephrectomy or cryoablation, using branching prompt logic to dynamically tailor queries based on prior LLM outputs for each note. Fifteen variables spanning pathology, radiology, and operative domains were extracted and compared to five manually-abstracted institutional datasets. In cases of disagreement, a second reviewer assessed the true value to measure not just agreement, but also accuracy. The cohort had a mean age of 62.8 years, was predominantly male (63%), primarily white (84%), and non-Hispanic (92%). Partial (57%) and radical (32%) nephrectomies were most common, with clear cell histology observed in 51% of cases. The LLM demonstrated strong extraction performance, with agreement rates ranging from 74.20% to 99.46% (Figure 1). Variables such as rhabdoid features, sarcomatoid features, and solitary kidney presence achieved the highest agreement, while ischemia temperature showed the lowest. For radiologic tumor size, LLM-extracted values correlated well with manual measurements from the image itself (ρ = 0.793). Manual adjudication showed the LLM was more accurate than human abstraction in 10 of 14 variables (Figure 2). This study demonstrates the feasibility of using a low-cost, open-source LLM for automated, PHI-safe data extraction in oncology. Continued development and multi-institutional validation will support broader deployment across cancer research and clinical practice.



# Abstract: Real World Evidence of Belzutifan Use in Patients with Von Hippel-Lindau (VHL) Associated Renal Cell Carcinoma (RCC), CNS Hemangioblastoma (CNS-Hb), or Pancreatic Neuroendocrine Tumors (pNETs)

**LEAD AUTHOR**

Narayan, Vivek K., University of Pennsylvania, Perelman School of Medicine, Philadelphia, PA, USA

**CO-AUTHORS**

Rimas V. Lukas, Northwestern University, Department of Neurology, Chicago, IL, USA

Jonathan Freimark, Analysis Group, Inc., Boston, MA, USA

Yan Song, Analysis Group, Inc., Boston, MA, USA

Travis Wang, Analysis Group, Inc., Boston, MA, USA

Zhuo Chen, Analysis Group, Inc., Boston, MA, USA

Joanne Chukwueke, Analysis Group, Inc., Boston, MA, USA

Murali Sundaram, Merck & Co., Inc., Rahway, NJ, USA

Rodolfo Perini, Merck & Co., Inc., Rahway, NJ, USA

Reshma Shinde, Merck & Co., Inc., Rahway, NJ, USA

Background:

Belzutifan is FDA-approved for VHL-associated RCC, CNS-Hb, and pNETs. This study evaluated belzutifan’s treatment patterns and impact on tumor reduction procedures (TRPs) in patients with VHL-associated RCC, CNS-Hb, or pNETs.

Methods:

Adults with VHL-associated RCC, CNS-Hb, or pNETs initiating belzutifan were identified from the Komodo Research Data claims database (2016-2023). The index date was the first belzutifan prescription. Time on belzutifan and proportion of days covered (PDC) were analyzed using different discontinuation gaps between prescriptions (≥30, ≥60, ≥90, or ≥120 days). Median time on belzutifan was estimated using Kaplan-Meier analysis. Monthly TRP incidence rates during the 2-year pre- and post-index periods were compared using generalized linear mixed models with random effects, adjusted for age, sex, geographic region, and insurance plan.

Results:

The analysis included 106 patients (mean age 41.5 [SD 13.2] years; 52.8% white; 81.1% commercially insured). RCC, CNS-Hb, and pNET were present among 88.7%, 51.9%, and 4.7% of patients, respectively. Median time on belzutifan was 14.6 months using a ≥30-day gap to define discontinuation. The median was not reached when using ≥60, ≥90, or ≥120-day gaps. Depending on the length of discontinuation gap, at 12 months, 61%-88% of patients remained on belzutifan, with PDC ranging from 84%-91%. Post-initiation, the rate for any TRP declined by 68% (IRR [95% CI] = 0.32 [0.18, 0.55], p<0.01), with 66% and 64% reductions in surgery for cerebellar/spinal hemangioblastomas and retinal laser therapy, respectively (both p <0.05) – Figure 1 shows the TRP distribution during the preand post-index periods.

Conclusions:

Belzutifan demonstrated a high treatment continuation rate and adherence, along with significantly reduced TRP rates, supporting its real-world effectiveness in managing VHL-associated RCC, CNS-Hb, and pNETs.



# Abstract: A multi-institution analysis of outcomes for 190 patients with metastatic chromophobe renal cell carcinoma with and without sarcomatoid features

**LEAD AUTHOR**

Doshi, Sahil, Memorial Sloan Kettering Cancer Center

**CO-AUTHORS**

Nazli Dizman, MD Anderson Cancer Center Andrea Knezevic, Memorial Sloan Kettering Cancer Center

Javier Gavira, Gustave Roussy

Pablo Barrios, Dana Farber Cancer Institute Zeynep Zengin, Yale Cancer Center

Ardit Feinaj, Johns Hopkins Medicine Hal Rives, Fox Chase Cancer Center

Nicolas Sayegh, UT Southwestern Medical Center

Charbel Hobeika, Cleveland Clinic

Mariangela Calabrese, Gustave Roussy

Reza Alaghehbandan, Cleveland Clinic

David Braun, Yale Cancer Center

Hans Hammers, UT Southwestern Medical Center

Matthew Zibelman, Fox Chase Cancer Center

Moshe Ornstein, Cleveland Clinic

Yasser Ged, Johns Hopkins Medicine

Andrew Hahn, MD Anderson Cancer Center

Bradley McGregor, Dana Farber Cancer Institute

Laurence Albiges, Gustave Roussy

Pavlos Msaouel, MD Anderson Cancer Center

Martin Voss, Memorial Sloan Kettering Cancer Center

Background: A subset of chromophobe renal cell carcinomas (ChRCC) harbor sarcomatoid features (SF), a marker of high-grade de-differentiation that universally confers poor prognosis. Whether presence of SF is predictive for response to immunotherapy (IO), an association that is well established in clear cell RCC, is unknown.

Methods: ChRCC patients treated systemically for metastatic disease at 9 academic centers were retrospectively reviewed. Presence or absence of SF was determined by local pathology report. First-line treatment regimens were categorized into IO-containing and non-IO. Time to treatment failure (TTF) and overall survival (OS) were estimated with the Kaplan-Meier method. Cox proportional hazards models were used to test the univariable effect of SF and the interaction between 1) IO vs non-IO therapies and 2) SF vs no SF.

Results: 190 patients with metastatic ChRCC were included; 27% (N=52) harbored SF. Median follow-up for survivors was 53 months (95% CI: 45, 70). TTF and OS data is summarized in the table. Across all patients, OS was superior in those with no SF vs SF (HR 0.38, 95% CI: 0.26-0.58; p<0.001). Among those with SF, patients treated with IO regimens (n=23) had statistically significantly superior OS compared to those with non-IO regimens (n=29; HR 0.29, 95% CI: 0.14-0.61; p=0.001). Among patients without SF, treatment with IO regimen (n=45) versus non-IO (n=93) had an inconclusive effect on OS (HR 0.85, 95% CI: 0.49-1.47; p=0.56). The degree of IO benefit was greater in the SF group (Cox interaction p=0.02).

Conclusions: We found that patients with SF had a larger treatment benefit with IO regimens than those without SF. These findings suggest that SF may not only have a prognostic significance but could be a predictive biomarker for IO therapy in metastatic ChRCC. This should be further explored, with implications for treatment selection and clinical trial design.



# Abstract: Fumarate hydratase variant prevalence in a reproductive carrier screening population

**LEAD AUTHOR**

Shuch, Brian, UCLA Institute of Urologic Oncology, Los Angeles, CA

**CO-AUTHORS**

Madeleine Armer-Cabral, Natera, Inc., Austin, TX

Julie Agapite, Natera, Inc., Austin, TX

Zuhal Ozcan, Natera, Inc., Austin, TX

Cooper Hall, Natera, Inc., Austin, TX

Kathleen Kueck, Natera, Inc., Austin, TX

Selina Wu, University of California, Los Angeles, Los Angeles, CA

Jeffrey Dungan, Natera, Inc., Austin, TX

Background

Germline variants in fumarate hydratase (FH) are associated with autosomal dominant (AD) hereditary leiomyomatosis and renal cell cancer (HLRCC), autosomal recessive (AR) fumarase deficiency (FMRD), or rarely both. Recent non-population-based studies suggest a carrier frequency for AD variants of ~1/1500. Assessing the carrier frequency of HLRCC- and FMRD-associated pathogenic/likely pathogenic (P/LP) variants in a reproductive carrier screening (RCS) population provides an additional estimate of the population incidence of this hereditary cancer syndrome.

Methods

A retrospective study of patients aged > 18 years receiving RCS from 01/2020-08/2024 was performed. FH variants were categorized into AD HLRCC variants and AR FMRD variants using data from external databases, internal sources, and primary literature. Variants associated with only AD HLRCC or AR FMRD were categorized definitively. For variants associated with both, the predominantly reported phenotype was chosen. Variants not previously reported were categorized by similarity to P/LP variants reported in the literature with associated phenotype. Overall carrier frequencies of both variant types were calculated. Carrier frequencies were stratified by race and ethnicity.

Results

300,686 individuals were included in the analysis. FH variants were detected in 0.32% of individuals in the cohort (AD HLRCC: 0.03% or 1/3712; AR FMRD: 0.30% or 1/339). Two variants were associated with AD and AR phenotypes (0.003%). Individuals of Ashkenazi Jewish descent had the highest incidence of AR variants (1.0%). White and Asian individuals had the highest incidence of AD variants (0.034%; 0.027%).

Conclusions

The high frequency of AD HLRCC variants identified through RCS aligns with recent estimates. An RCS population may be a better estimate of the population-based incidence of FH variants, which may be more prevalent than previously recognized.

# Abstract: Long-term exposure to ambient fine particulate matter and risk of renal cell cancer in the NIH-AARP Diet and Health Study

**LEAD AUTHOR**

Li, Jinhui, Stanford University

**CO-AUTHORS**

Zhengyi Deng, Stanford University

Minji Jung, Stanford University

Andres Cardenas, Stanford University

John Leppert, Stanford University

Benjamin Chung, Stanford University

Marvin Langston, Stanford University

Background: Ambient fine particulate matter (PM2.5) has been linked with elevated risk of some cancers, but the evidence for renal cell carcinoma (RCC) incidence remains limited and inconclusive.

Methods: We investigated the association between historical exposure of PM2.5 and RCC risk for the first time in the U.S. large Cohort, NIH-AARP Diet and Health Study, among 497,987 participants from six states and two metropolitan. Time-varying Cox proportional hazards model using calendar year as time scale was applied to estimate the RCC incidence associated with per 5 μg/m3 increase in PM2.5 exposure was defined as the 5-year average concentration with a 10-year lag prior to the outcome assessment. We also assessed PM2.5 interactions with hypothesized effect modifiers, and further explored the effects of PM2.5 exposure among individuals with diabetes and hypertension.

Results: 3359 incident RCC cases were identified through follow-up to 2018. A non-significantly increased risk of RCC associated with per 5 ug/m3 increase in PM2.5 exposure (HR = 1.03, 95% CI: 0.97-1.10). Participants aged over 65 years at enrollment had an increased risk of RCC versus other age groups (HR = 1.06, 95% CI: 0.96-1.19; p-interaction = 0.004) and risk was increased among participants with diabetes versus those without diabetes with marginal significance (HR = 1.20, 95% CI: 0.98-1.48; p-interaction = 0.022). Notably, PM2.5 exposure was associated with a significantly higher incidence of RCC in the participants who were current smokers (HR = 1.58, 95% CI: 1.06-2.36; p-interaction = 0.014) among all the participants with hypertension.

Conclusion: In this large U.S. cohort with historical ambient air pollution estimates, long-term exposure to PM2.5 was potentially associated with an elevated risk of RCC. If confirmed in future studies, these findings suggest the importance of considering comorbidities and investigating specific PM2.5 components and co-exposure to other air pollutants in modifying RCC risk.

# Abstract: A phase 1/2, open label, single arm study on safety, tolerability and anti-tumor efficacy of orellanine treatment in patients with metastatic clear-cell renal cell carcinoma (ccRCC) or papillary renal cell carcinoma (pRCC)

**LEAD AUTHOR**

Tannir, Nizar, M., University of Texas MD Anderson Cancer Center, Houston, TX

**CO-AUTHORS** Adanma Ayanambakkam, OU Health Stephenson Cancer Center, Oklahoma City, OK

Laurence Albiges, Institut Gustave Roussy, Paris, France

Arpita Desai, University of California, San Francisco, CA

Saby George, Roswell Park Comprehensive Cancer Center, Buffalo, NY

André Mansinho, START Lisbon, Lisbon, Portugal

Nataliya Mar, University of California, Irvine, CA

Melissa Reimers, Washington University, St. Louis, MO

Sandy Srinivas, Stanford University Medical Center, San Francisco, CA

Karine Tawagi, UI Health, Chicago, IL

Jeffrey Yachnin, Karolinska University Hospital, Stockholm, Sweden

Yousef Zakharia, Mayo Clinic Arizona, Phoenix, AZ

Börje Haraldsson, University of Gothenburg and Oncorena AB, Sweden

Background: Immune checkpoint inhibitors (ICIs) and targeted agents are standard-of-care (SOC) for advanced renal cell carcinoma (RCC) and have significantly improved survival. However, novel therapies are urgently needed for patients who are refractory to SOC or relapse after an initial response—particularly those requiring chronic hemodialysis due to treatment-related nephrotoxicity, or comorbidities. This subgroup faces limited treatment options and is often excluded from clinical trials.

ONC175 (orellanine) is a first-in-class, highly kidney-specific cytotoxin derived from Cortinarius mushrooms. Accidental ingestion in humans causes irreversible renal injury without major systemic toxicity. Preclinical studies demonstrate that ONC175 induces apoptosis and ferroptosis in clear cell (ccRCC) and papillary RCC (pRCC), achieving >90% tumor volume reduction in xenograft models. Its tumor selectivity is mediated by identified orellanine transporters, Fig. 1. It is a first-in-class, small (252 Dalton), water-soluble molecule, that may be classified under L01CX. In March 2025, FDA approved the IND (167761).

Methods: Oncorella-1 (NCT05287945) is a multi-part, adaptive trial enrolling patients with metastatic ccRCC or pRCC on maintenance hemodialysis and progressive disease following SOC therapies (≥6 months since last ICI).

- Part A: Inpatient dose-escalation in 5 patients at a single site—completed.
- Part B: Ongoing dose-exposure expansion in 20–25 patients across multiple sites to define the recommended Phase 2 dose (RP2D).
- Part C: Planned dose-expansion based on Part B findings.

Following a 30-min IV infusion of ONC175, patients undergo 1–3 days of drug exposure with intensive monitoring. Primary endpoints include safety, tolerability, and pharmacokinetics; secondary endpoints include preliminary antitumor activity and dialysis-specific safety parameters.

This trial addresses a rare and underserved patient population with advanced RCC on dialysis, for whom no approved systemic therapies currently exist.

Figure 1. Orellanine selectively targets renal cell carcinoma through specific solute carriers expressed on RCC and the proximal tubular cells they originate from



# Abstract: Safety and efficacy of HLA-G–targeted CAR T cell therapy (IVS-3001) in patients with advanced clear cell RCC and other HLA-G– positive solid tumors: Clinical trial in progress

**LEAD AUTHOR**

Tannir, Nizar, MD Anderson Cancer Center

**CO-AUTHORS**

Samer Ali Srour, MD Anderson Cancer Center

Amir A. Jazaeri, MD Anderson Cancer Center

Matthew T. Campbell, MD Anderson Cancer Center

Yago Nieto, MD Anderson Cancer Center

Cara L Haymaker, MD Anderson Cancer Center

Ying Yuan, MD Anderson Cancer Center

Israa Salih, MD Anderson Cancer Center

Yali Yang, MD Anderson Cancer Center

Vale´rie Doppler, MD Anderson Cancer Center

Julie Garibal, MD Anderson Cancer Center

Marie Escande, MD Anderson Cancer Center

Qi Melissa Yang, MD Anderson Cancer Center

Jake Kushner, MD Anderson Cancer Center

Jane Koo, MD Anderson Cancer Center

Serdar A Gurses, MD Anderson Cancer Center

Ann Cimo, MD Anderson Cancer Center

David S. Hong, MD Anderson Cancer Center

Siging Fu, MD Anderson Cancer Center

Funda Meric-Bernstam, MD Anderson Cancer Center

Aung Naing, MD Anderson Cancer Center

Background: Immunotherapies have transformed cancer treatment, yet only a minority of patients experience durable responses. IVS-3001 is an innovative autologous chimeric antigen receptor (CAR) T-cell therapy specifically targeting Human Leukocyte Antigen (HLA-G). HLA-G is an immune-modulatory checkpoint molecule expressed on various solid tumors, positioning it as an ideal a tumor-specific targeted antigen. Our third-generation CAR construct features enhanced T cell activation and persistence against HLA-G. By harnessing IVS-3001 to target HLAG and revitalize immune cells, we aim to overcome the suppressive tumor microenvironment and improve antitumor activity, potentially leading to better outcomes for patients with advanced solid tumors who otherwise have no standard options known to confer clinical benefit.

Methods: Study NCT05672459 is a First-in-Human, phase 1/2a, safety and efficacy study of IVS-3001 in subjects with previously treated advanced HLA-G-positive solid tumors. Phase 1 (n = 24 patients) is a Bayesian Optimal Interval Design (BOIN) with primary objective to determine the safety, tolerability and the recommended phase 2 dose. The primary objective for phase 2 (n = 90 patients) is to evaluate the anti-tumor activity of IVS-3001. The secondary objectives of the study are to evaluate i) pharmacokinetic profile of IVS-3001 (persistence, expansion); ii) the clinical activity of IVS-3001 in selected HLA-G+ solid tumor types; iii) assess the long-term safety of IVS-3001. Exploratory endpoints include functionality of CAR-T cells, immune biomarker changes, and relationships with clinical response. Key inclusion criteria include adults with advanced clear cell RCC and solid tumors expressing HLA-G; ECOG PS 0-1; adequate organ function. Key exclusion criteria comprise uncontrolled brain metastasis and prior exposure to HLA-G targeted therapy. Subjects undergo lymphodepletion with fludarabine and cyclophosphamide on days -5 to -3, followed by CAR-T cell infusion on day 0 and a 28-day monitoring period for dose limiting toxicity. Response assessment is per RECIST. Study is currently accruing at Dose level 4. Active recruitment and enrollment are ongoing at The University of Texas MD Anderson Cancer Center, Houston, Texas. Clinical trial information: NCT05672459.

Research Sponsor/Funding: Invectys Inc. IVS 3001 program received funding from Cancer Prevention & Research Institute of Texas (CPRIT) under grant DP200034

# Abstract: The Translocation Renal Cell Carcinoma Project: Direct Digital Engagement of Patients with a Rare Kidney Cancer

**LEAD AUTHOR**

Diehl, Diane M., Broad Institute

**CO-AUTHORS**

Zoe Geary, Broad Institute

Elana Anastasio, Broad Institute

Parker Chastain, Broad Institute

Melissa Chiumiento, Broad Institute

Taisha Hendrickson, Broad Institute

Cheryl Henrick, Broad Institute

Jordan Doucette, Broad Institute

Eirian Siegal-Botti, Broad Institute

Jeremy Johnson, Broad Institute

Ashley Matthews, Broad Institute

Noriela Elia, Broad Institute

Beena Thomas, Broad Institute

Elizabeth A. Mullen, Dana-Farber/Boston Children's Blood Disorders and Cancer Center

Srinivas R. Viswanathan, Dana-Farber Cancer Institute

Translocation renal cell carcinoma (tRCC)—also known as TFE3/TFEB-rearranged RCC or Xp11.2 translocation RCC—is a rare form of kidney cancer with very limited tumor tissue available for research.

To help address this gap, we launched a tRCC research initiative in March 2025, led by Dr. Srinivas Viswanathan, PhD at Dana-Farber Cancer Institute and Dr. Elizabeth Mullen, at Dana-Farber/Boston Children's Hospital. We believe that every patient with tRCC, from all treatment centers in the US and Canada, should have the opportunity to contribute to research to improve understanding and treatment options for this cancer.

Our study aims to:

Define the molecular landscape of tRCC (genetic mutations andcopy number alterations)

Define transcriptional subtypes of tRCC and associations withdifferent fusions (e.g. TFE3 vs TFEB)

Identify correlates of response to various systemic therapies formetastatic tRCC

Identify genetic changes in response to treatment (throughcollecting metastatic/post-treatment samples)

Determine correlates of recurrence after kidney resection forpatients with localized disease

Determine differences in molecular landscape between pediatricand adult tRCC

Anyone in the US or Canada with a diagnosis of tRCC is eligible to participate. So far, 22 participants from across the US have enrolled. We are deeply grateful to our clinical collaborators, advocacy partners, and the tRCC community for helping share awareness of this opportunity.

Ongoing work includes:

Medical record acquisition and abstraction Sequencing (FFPE, saliva, blood): WGS, RNA-seq, Single-Cell RNASeq Diagnostic journey surveys Legacy project to enroll children who have passed away



# Abstract: Association between wildfire-dominated PM2.5 exposure and renal cell carcinoma survival in California

**LEAD AUTHOR**

Gulati, Shuchi, 1. Division of Hematology/Oncology, Department of Internal Medicine, UC Davis School of Medicine, Sacramento, California 2. University of California Davis Comprehensive Cancer Center, Sacramento, California

**CO-AUTHORS** Mariela Alaniz, 3. Department of Public Health Sciences, UC Davis School of Medicine, University of California, Davis

Sean Raffuse, 4. Air Quality Research Center, University of California Davis, Davis, California 5. Environmental Health Sciences Center, University of California Davis, Davis, California

Irva Hertz-Picciotto, 3. Department of Public Health Sciences, UC Davis School of Medicine, University of California, Davis 5. Environmental Health Sciences Center, University of California Davis, Davis, California

Primo N. Lara, Jr, 1. Division of Hematology/Oncology, Department of Internal Medicine, UC Davis School of Medicine, Sacramento, California 2. University of California Davis Comprehensive Cancer Center, Sacramento, California

Theresa Keegan, 1. Division of Hematology/Oncology, Department of Internal Medicine, UC Davis School of Medicine, Sacramento, California 2. University of California Davis Comprehensive Cancer Center, Sacramento, California 3. Department of Public Health Sciences, UC D

Shehnaz Hussain, 2. University of California Davis Comprehensive Cancer Center, Sacramento, California 3. Department of Public Health Sciences, UC Davis School of Medicine, University of California, Davis

Background: Wildfire emissions, rich in fine particulate matter (PM2.5), are known to affect respiratory cancers; however, their role in renal cell carcinoma (RCC) is unclear. We evaluated the impact of wildfire-dominated PM2.5exposure on RCC survival in California, where wildfires have recently become frequent and devastating.

Methods: RCC patients diagnosed between 2017-2019 from California Cancer Registry- who survived at least 12 months post-diagnosis and had a known residential address, were included. PM2.5 exposure was estimated using validated random forest regression over a 1-km^2^ spatial resolution and linked to patient residence (±1 km). Exposure metrics included mean annual PM2.5 and days with PM2.5 concentrations ≥9, 20, 35, and 55 µg/m^3^ during the first year after diagnosis. Thresholds ≥35 and ≥55 µg/m^3^ were considered markers of wildfire-dominant exposure. Cox-proportional hazards regression was used to estimate hazard ratio (HR) for all-cause mortality associated with PM2.5; stratified by smoking, systemic therapy, and BMI.

Results: Among 14,759 RCC patients (mean age: 63 years; 64% male; 51% non-Hispanic White), a 10 µg/m^3^ increase in mean annual PM2.5 was associated with a 22.1% increased hazard of death (HR 1.22; 95% CI 0.98–1.53). Risk was higher among patients receiving systemic therapy (HR 1.62), with BMI <30 (HR 1.44), and never-smokers (HR 1.34) (Table). Notably, additional 10 days with PM2.5 ≥55 µg/m^3^ was associated with a 3.4% reduced hazard (HR 0.97; CI 0.86–1.09). Stage IV patients with <10 days of PM2.5≥20 µg/m^3^ had significantly longer 25% quartile survival than those with >10 days (10.1 vs. 8.3 months, p=0.048).

Conclusions: Post-diagnosis exposure to higher PM2.5 is independently associated with worse survival in RCC patients. As wildfires intensify, environmental health interventions for newly diagnosed RCC patients are crucial, and molecular studies are needed to uncover underlying mechanisms.



# Abstract: Spontaneous Regression of Biopsy-Confirmed Renal Cell Carcinoma Primary Tumors on Active Surveillance- Clinical and Pathologic Correlates

**LEAD AUTHOR**

Lotan, Paz, University of Wisconsin, School of Medicine and Public Health, Madison, WI, USA

**CO-AUTHORS**

Zhe Jing, Roswell Park Cancer Center, Buffalo, NY

Shervin Badkhshan, Roswell Park Cancer Center, Buffalo, NY

Arun Menon, Roswell Park Cancer Center, Buffalo, NY

Amandip Cheema, Roswell Park Cancer Center, Buffalo, NY

Muammer Altok, Roswell Park Cancer Center, Buffalo, NY

Bo Xu, Roswell Park Cancer Center, Buffalo, NY

Michael Petroziello, Roswell Park Cancer Center, Buffalo, NY

Charles Roche, Roswell Park Cancer Center, Buffalo, NY

Ahmed A. Hussein, Roswell Park Cancer Center, Buffalo, NY

Eric C. Kauffman, Roswell Park Cancer Center, Buffalo, NY

Background: Partial or complete spontaneous regression (SR) of renal primary tumors has received little academic attention to date, due to its suspected infrequency and presumed underlying benign pathology or potential association with radiographic imaging limitations. This study aimed to characterize the incidence, clinical features, and prognostic significance of SR in the largest series to our knowledge of histologically confirmed renal cell carcinoma (RCC) primary tumors managed with active surveillance (AS).

Methods: Using a prospectively maintained renal mass AS registry at a National Comprehensive Cancer Network center, we retrospectively reviewed all patients with biopsy-confirmed RCC and primary tumor size documented in three dimensions on >/=2 cross-sectional scans obtained >/=6 months apart. Tumor volume was calculated as an ellipsoid (0.52xD1xD2xD3), and SR was defined as >30% volume reduction. Two radiologists independently reviewed SR cases to confirm regression and rule out tissue aspiration during biopsy. A consecutive subset of 20 SR cases underwent semi-automated volume quantification using volumetry software (Myrian, Inc.), followed by Spearman correlation with manually estimated volumes. Univariate and multivariable analyses identified variables associated with SR.

Results: Of 182 patients with biopsy-confirmed RCC tumors on AS for a median of 31 months, 48 (26%) experienced SR, including a median volume reduction of 60%. Strong correlation was observed between manually estimated and volumetrically measured regression magnitudes (Figure 1A). Chromophobe and papillary subtypes exhibited significantly higher SR rates and greater regression magnitudes compared to clear cell. On multivariate analysis, non-clear cell histology (OR 4.63, p<0.001) and tumor multifocality (OR 2.84, p=0.028) were independently associated with SR. SR was also associated with longer delayed intervention-free survival (Figure 1B).

Conclusion: A subset of RCC patients undergoing AS experience SR with associated prognostic advantage. Better understanding of this phenomenon will facilitate optimal risk stratification for AS management and may help uncover novel therapeutic strategies.



# Abstract: Multi-institution Evaluation of Patient versus Urologist Rated Performance Status and Association with Renal Cell Carcinoma Surgical Outcomes

**LEAD AUTHOR**

Lotan, Paz, University of Wisconsin, School of Medicine and Public Health, Madison, WI, USA

**CO-AUTHORS**

E. Jason Abel, The University of Wisconsin, School of Medicine and Public Health, Madison, WI, USA

Viraj A. Master, Emory University, Atlanta, GA

Danny Roadman, The University of Wisconsin, School of Medicine and Public Health, Madison, WI, USA

Gregory Palmateer, Emory University, Atlanta, GA

Dattatraya Patil, Emory University, Atlanta, GA

Glenn O. Allen, The University of Wisconsin, School of Medicine and Public Health, Madison, WI, USA

Kye Nichols, The University of Wisconsin, School of Medicine and Public Health, Madison, WI, USA

David F. Jarrard, The University of Wisconsin, School of Medicine and Public Health, Madison, WI, USA

Elizabeth Koehne, The University of Wisconsin, School of Medicine and Public Health, Madison, WI, USA

Michael C. Risk, The University of Wisconsin, School of Medicine and Public Health, Madison, WI, USA

Kyle A. Richards, The University of Wisconsin, School of Medicine and Public Health, Madison, WI, USA

Daniel D. Shapiro, The University of Wisconsin, School of Medicine and Public Health, Madison, WI, USA

Background: ECOG performance status (PS) scale is associated with treatment outcomes and commonly used for risk stratification. Physicians generally determine PS, although recent studies suggest that patient self-reported PS (PrPS) may differ from physician assessment and is independently associated with treatment outcomes. This study aimed to determine concordance between urologist-reported PS (UrPS) and PrPS and evaluate associations with postoperative complications among patients undergoing surgery for renal cell carcinoma (RCC).

Methods: Patients with RCC were enrolled between 2021 and 2025 at two institutions. During pre-surgical clinic visits, patients self-reported their ECOG PS using paper surveys before meeting the urologist, who independently assessed ECOG PS blinded to the patient’s self-report. Agreement between reports was evaluated using weighted kappa statistics. Univariate and multivariable analyses were used to identify factors associated with disagreement and associations with 90-day postoperative major complications (Clavien-Dindo grade ≥3a), adjusting for potential confounders.

Results: Overall, 238 patients were enrolled. 90-day major complications were identified in 20/238 (8.4%) (Fig.1A). PrPS and UrPS were concordant in 66% of patients (kappa= 0.32). Urologists reported better and worse PS in 21% and 13% of patients, respectively (Fig.1B). Urologists rarely reported ECOG≥2 compared to patients (4% vs. 10% of cases, respectively, p=0.01). Patients self-reporting worse ECOG scores than urologists had higher-stage disease (p=0.047) and were younger (p=0.02).

After adjusting for age, sex, Charlson comorbidity ≥3, BMI, and stage, both UrPS and PrPS were associated with major complications (OR 2.36; p=0.02 and OR 1.82; p=0.02, respectively). However, for ECOG ≥2, only the PrPS remained statistically significant (OR 4.45, p=0.01).

Conclusions: ECOG PS reporting is frequently discordant between urologists and patients. While both PrPS and UrPS are associated with risk of major complication, urologists rarely report poor PF (ECOG≥2), and patients reporting ECOG≥2 should be considered at high risk for major complication.



# Abstract: AI-empowered Cellular Morphometric Biomarkers predict treatment response and prognosis in clear cell renal cell carcinoma

**LEAD AUTHOR**

Gulati, Shuchi, Department of Internal Medicine, Division of Hematology and Oncology, University of California Davis, Sacramento, CA, United States

**CO-AUTHORS**

Dan Li, Key Laboratory of Cancer Prevention and Intervention, China National Ministry of Education, Key Laboratory of Molecular Biology in Medical Sciences, Hangzhou, ZJ, China; Department of Medical Oncology, The Second Affiliated Hospital, Zhejiang University

April Mao, Department of Mathematics, University of California, Los Angeles, Los Angeles, CA, United States

Dawei Wang, Biological Systems and Engineering Division, Lawrence Berkeley National Laboratory, Berkeley, CA, United States; Berkeley Biomedical Data Science Center, Lawrence Berkeley National Laboratory, Berkeley, CA, United States

Ryan Hong, Biological Systems and Engineering Division, Lawrence Berkeley National Laboratory, Berkeley, CA, United States; Berkeley Biomedical Data Science Center, Lawrence Berkeley National Laboratory, Berkeley, CA, United States

Jesus Pérez-Losada, Instituto de Biología Molecular y Celular del Cáncer (IBMCCCIC), Universidad de Salamanca/CSIC, Salamanca, 37007, Spain; Instituto de Investigación Biosanitaria de Salamanca (IBSAL), Salamanca, 37007, Spain

Kuang-Yu Jen, Department of Pathology and Laboratory Medicine, University of California, Davis School of Medicine, Sacramento, CA, United States

Jian-Hua Mao, Biological Systems and Engineering Division, Lawrence Berkeley National Laboratory, Berkeley, CA, United States; Berkeley Biomedical Data Science Center, Lawrence Berkeley National Laboratory, Berkeley, CA, United States

Ying Yuan, Key Laboratory of Cancer Prevention and Intervention, China National Ministry of Education, Key Laboratory of Molecular Biology in Medical Sciences, Hangzhou, ZJ, China

Kenneth Iczkowski, Department of Pathology and Laboratory Medicine, University of California, Davis School of Medicine, Sacramento, CA, United States

Hang Chang, Biological Systems and Engineering Division, Lawrence Berkeley National Laboratory, Berkeley, CA, United States; Berkeley Biomedical Data Science Center, Lawrence Berkeley National Laboratory, Berkeley, CA, United States

Background: Clear cell renal cell carcinoma (ccRCC) is histologically, molecularly, and clinically heterogeneous. Effective prognostication and treatment prediction require multi-modality biomarkers, as no single marker has proven sufficient.

Methods: We developed and validated an AI-based pipeline, Cellular Morphometric Biomarker via Machine Learning (CMB-ML), to extract and quantify 15 key morphometric features (e.g., nuclear size, chromatin density, nuclear contour smoothness) from H&E-stained whole slide images (WSIs). These features were used to define 73 cellular morphometric biomarkers (CMBs), from which CMB risk groups (CMBRG) were derived. The pipeline was applied to 490 ccRCC cases from the TCGAKIRC cohort for model development. Three validation cohorts were used: CPTACCCRCC (public), ZJU-CCRCC, and UCD-CCRCC (independent hospital cohorts). The study aimed to evaluate the prognostic and predictive potential of CMBRG in ccRCC.

Results: CMBRG stratified patients into CMB-low (better prognosis) and CMB-high (worse prognosis) groups with significantly different overall survival in TCGA (p<0.0001), CPTAC (p=0.0039), and ZJU (p=0.023) cohorts and remained an independent prognostic factor after adjusting for clinical variables (e.g., Stage, Age, Sex). Proteomic pathway analysis (CPTAC) revealed enrichment of mTOR and HIF-1 pathways in the CMB-low group, while glycolysis and fatty acid metabolism were enriched in the CMB-high group. The CMB-high group was more inflamed (higher CD8+ T cell signature), with higher expression of immune-suppressive signatures (MDSCs, Tregs, IL6-JAK-STAT). Response to mTOR inhibitors was confirmed in the CMB-low patients in a UC Davis ccRCC cohort, where the risk score correlated negatively with treatment duration (p=0.038) and differed significantly between responders and non-responders (p=0.025). Somatic mutations (VHL, BAP1, PBRM1, SETD2), tumor mutational burden, and neoantigen load did not differ significantly between CMB-high or low groups.

Conclusion: CMBRG adds prognostic and predictive value in ccRCC beyond standard clinical and pathological markers. Further validation in response to HIF2α and immune checkpoint inhibitors is ongoing.



# Abstract: A decision-making tool for first-line treatment selection in metastatic renal cell carcinoma based on plasma proteomics

**LEAD AUTHOR**

Steiner, Clara, Department of Medical Oncology, Dana-Farber Cancer Institute, Boston, MA, USA

**CO-AUTHORS**

Coren Lahav, Bioinformatics, OncoHost, Binyamina-Giv'at Ada, Israel

Yehonatan Elon, Physics, OncoHost, Binyamina-Giv'at Ada, Israel

Adam P. Dicker, Department of Radiation Oncology, Thomas Jefferson University, Philadelphia, PA, USA

Michal Harel, R&D, OncoHost, Binyamina-Giv'at Ada, Israel

Nili Dahan, Diagnostics, OncoHost, Binyamina-Giv'at Ada, Israel

Gil Loewenthal, R&D, OncoHost, Binyamina-Giv'at Ada, Israel

Liliana Ascione, Department of Medical Oncology, Dana-Farber Cancer Institute, Boston, MA, USA

Marc Machaalani, Department of Medical Oncology, Dana-Farber Cancer Institute, Boston, MA, USA

Eddy Saad, Department of Medicine, Beth Israel Deaconess Medical Center, Boston, MA, USA

Renée Maria Saliby, Internal Medicine, Yale University School of Medicine - Yale Cancer Center, New Haven, CT, USA

Marc Eid, Department of Medical Oncology, Dana-Farber Cancer Institute, Boston, MA, USA

Karl Semaan, Department of Medicine, Brigham and Women's Hospital, Boston, MA, USA

François-Xavier Danlos, Drug Development Department - LRTI, Institut Gustave Roussy, Villejuif, France

Laurence Albiges, Medical Oncology Department, Institut Gustave Roussy, Villejuif, France

Toni K. Choueiri, Department of Medical Oncology, Dana-Farber Cancer Institute, Boston, MA, USA

Wenxin Xu, Department of Medical Oncology, Dana-Farber Cancer Institute, Boston, MA, USA

Background: Treatment options for metastatic renal cell carcinoma (mRCC) include immune checkpoint inhibitors (ICI), tyrosine kinase inhibitors (TKI), and combination therapies. However, optimal therapy selection is challenging. Here, we present a novel plasma proteomics-based model to inform first-line treatment decisions in mRCC.

Methods: Baseline plasma samples were obtained from 178 mRCC patients at two academic centers receiving TKI monotherapy (n=62), ICI monotherapy or ICI-ICI combination therapy (n=76), or ICI-TKI combinations (n=40). Deep plasma proteomic profiling was performed using an aptamer-based technology. A machine-learning model to predict TKI response was developed using cross-validation (CV, n=119) on the TKI monotherapy group after selecting proteins with predictive capabilities exclusively in this group. The model output stratified first-line patients into PRO-TKI and PRO-ICI patients. The model's predictive performance was evaluated with Kaplan-Meier plots and multivariate Cox analyses for overall survival (OS) in an independent validation cohort (n=59).

Results: In the TKI and ICI-TKI acohorts, PRO-TKI patients had significantly longer OS than PRO-ICI patients. However, in the ICI-ICI cohort, OS was similar in PRO-TKI and PRO-ICI groups, suggesting that the model is predictive for TKI-containing regimens (Table 1). In the independent validation cohort, PRO-TKI patients had superior OS with ICI-TKI compared to ICI-ICI (HR=0.14, p-value = 0.003). In contrast, PRO-ICI patients had inferior OS with ICI-TKI compared to ICI-ICI (HR=2.88, p-value = 0.11). A significant interaction was shown for treatment modality and model output (HR = 0.47, p-value = 0.003).

Conclusions: We describe a predictive model for survival benefit from TKI-containing regimens in mRCC based on pre-treatment plasma proteomics. The model output can serve as a tool to support therapeutic decisions.

**Table 1:** Model output and OS

Patient treatment	n	Hazard ratio (HR)	95% confidence interval (CI)	p-value	Validation method	PRO-TKI mOS	PRO-ICI mOS
TKI	62	0.49	0.27-0.88	0.02	CV	57.5 months	25.5 months
ICI-TKI	40	0.11	0.03-0.44	<0.001	Independent	Not reached	15.1 months
ICI-ICI	19	2.23	0.26-2.54	0.28	Independent	20.7 months	33.3 months

# Abstract: Enriched YES1 tumor expression at the stromal interface is associated with worse overall survival in clear cell renal cell carcinoma when treated with first-line immune checkpoint blockade

**LEAD AUTHOR**

Obermayer, Alyssa, Department of Biostatistics and Bioinformatics, Moffitt Cancer Center

**CO-AUTHORS**

Alex Soupir, Department of Biostatistics and Bioinformatics, Moffitt Cancer Center

Carlos MoranSegura, Analytic Microscopy Lab, Moffitt Cancer Center

Natasha Francis, Tissue Core, Moffitt Cancer Center

Paola Echevarria, Nontherapeutic Research Operations, Moffitt Cancer Center

Doug Marchion, Tissue Core, Moffitt Cancer Center

Sean Yoder, Genomics Core, Moffitt Cancer Center

Jodi Balasi, Department of Pathology, Moffitt Cancer Center

Roy Elias, Department of Medical Oncology, Johns Hopkins University

Nirmish Singla, Department of Urology, Johns Hopkins University

Michelle Churchman, Aster Insights

Eric Singer, Division of Urologic Oncology, The Ohio State University Comprehensive Cancer Center

Sean Kern, Department of Urology, Uniformed Services University/Murtha Cancer Center

Yousef Zakharia, Department of Medical Oncology, University of Iowa

Paul Viscuse, Department of Medical Oncology, University of Virginia

Patrick Hensley, Department of Urology, University of Kentucky

Daniel Grass, Department of Genitourinary Oncology, Moffitt Cancer Center

Jasreman Dhillon, Department of Pathology, Moffitt Cancer Center

James Mulé, Department of Immunology, Moffitt Cancer Center

Brandon Manley, Department of Genitourinary Oncology, Moffitt Cancer Center

Tim Shaw, Department of Biostatistics and Bioinformatics, Moffitt Cancer Center

Mitchell Hayes, Department of Genitourinary Oncology, Moffitt Cancer Center

Background: Immune checkpoint blockade (ICB) has improved response rates for patients with advanced clear cell renal cell carcinoma (ccRCC). However, most ccRCC patients develop resistance. YES1 expression may be upregulated in malignant cells at the tumor-stromal interface of treatment resistant primary tumors. Our primary objective was to validate YES1 upregulation in an expanded cohort of ICB and tyrosine kinase inhibitor (TKI) exposed patients, develop a bulk RNA sequencing (RNAseq) gene signature, and test for overall survival (OS) in the Oncology Research Information Exchange Network’s (ORIEN) multi-institutional molecular database of patients treated with ICB.

Methods: Tissue microarrays were constructed using one-millimeter cores from the tumor-stromal interface of surgically excised primary ccRCC tumors and normal controls. Single-cell spatial transcriptomics (scST) was obtained using a 6,000 gene panel and CosMxTM spatial molecular imager. Malignant cell YES1 expression was compared between treatment-naïve and treatment-exposed samples. Same patient RNAseq data were used to construct a signature using genes that correlated with YES1 expression on scST. We tested OS in treatment-naive patients with a metastatic diagnosis who received a first-line ICB regimen in the multi-institutional ORIEN cohort. Cox regression was used and YES1 score was treated as a continuous variable.

Results: 175,838 cells were analyzed from 32 patients who were treatment-naïve or treatment-exposed (ICB only, TKI only, or ICB/TKI). On linear mixed effect modeling, YES1 expression was higher in ICB-exposed patients but not TKI-exposed patients (p = 0.003 and p = 0.856). 1919 bulk genes that correlated with single cell malignant YES1 expression were included in the YES1 score. 250 patients with treatment naïve ccRCC bulk RNA went on to receive a first-line ICB regimen in the ORIEN cohort. 72% of specimens were from primary tumors. High YES1 score was associated with worse OS on ICB (HR 1.77, CI 1.02-3.09, p = 0.04).

Conclusions: We found that a spatially informed bulk RNA gene signature associated with malignant cell YES1 upregulation at the tumor-stromal interface was associated with worse OS in a cohort of patients who received first-line ICB treatment.



# Abstract: Cluster of differentiation 70 (CD70) is associated with an angiogenic subtype in patients with metastatic renal cell carcinoma (mRCC)

**LEAD AUTHOR**

Abreu de Goes, Vitor, Department of Medical Oncology & Therapeutics Research, City of Hope Comprehensive Cancer Center, Duarte, CA

**CO-AUTHORS**

Nicholas J Salgia, Department of Immunology, Roswell Park Comprehensive Cancer Center, Buffalo, NY, USA

Yu Jun Li, Department of Medical Oncology & Therapeutics Research, City of Hope Comprehensive Cancer Center, Duarte, CA

Salvador Jaime-Casas, Department of Medical Oncology & Therapeutics Research, City of Hope Comprehensive Cancer Center, Duarte, CA

Miguel Zugman, Department of Medical Oncology & Therapeutics Research, City of Hope Comprehensive Cancer Center, Duarte, CA

Koral Shah, Department of Medical Oncology & Therapeutics Research, City of Hope Comprehensive Cancer Center, Duarte, CA

Daniela V. Castro, Department of Medical Oncology & Therapeutics Research, City of Hope Comprehensive Cancer Center, Duarte, CA

Xiaochen Li, Department of Medical Oncology & Therapeutics Research, City of Hope Comprehensive Cancer Center, Duarte, CA

Benjamin Mercier, Department of Medical Oncology & Therapeutics Research, City of Hope Comprehensive Cancer Center, Duarte, CA

Regina Barragan-Carrillo, Department of Medical Oncology & Therapeutics Research, City of Hope Comprehensive Cancer Center, Duarte, CA

Joann Hsu, Department of Medical Oncology & Therapeutics Research, City of Hope Comprehensive Cancer Center, Duarte, CA

Ruchi Agarwal, Department of Medical Oncology & Therapeutics Research, City of Hope Comprehensive Cancer Center, Duarte, CA

Hedyeh Ebrahimi, Department of Medical Oncology & Therapeutics Research, City of Hope Comprehensive Cancer Center, Duarte, CA

Alexander Chehrazi-Raffle, Department of Medical Oncology & Therapeutics Research, City of Hope Comprehensive Cancer Center, Duarte, CA

Sumanta Kumar Pal, Department of Medical Oncology & Therapeutics Research, City of Hope Comprehensive Cancer Center, Duarte, CA

Background CD70 is a transmembrane protein belonging to the tumor necrosis factor family, acting as a co-stimulator of immune cells. In RCC, CD70 overexpression and interaction with the CD27 receptor enables immune evasion and tumor progression. Thus, CD70 is under investigation as a target for chimeric antigen receptor-T (CAR-T) cell therapies and antibody drug conjugates (ADCs) (NCT05990621, NCT02216890). Here, we describe clinical outcomes and molecular patterns based on CD70 expression in mRCC. Methods We evaluated normalized transcript-per-million gene expression count matrices and clinical metadata from patients with mRCC. Data was retrieved from IMmotion151, HCRN-GU16-260, and Javelin101 cohorts, as well as from The Cancer Genome Atlas (TCGA). We stratified individuals into CD70 high and low expressors according to median expression per cohort. Non-negative Matrix Factorization (NMF) was used to define transcriptional clusters in both groups within IMmotion151. Fisher’s Exact Tests were employed to compare cluster abundance between CD70 groups. Results CD70 was overexpressed within tumor samples compared to individual-matched controls in TCGA (n=517). Using definitions from IMmotion151 (n=822), different proportions of NMF clusters were identified between CD70high and CD70low. Specifically, CD70high tumors were associated with angiogenic (p<0.00001) and complement/oxidation (p=0.003) subtypes (Figure 1). In contrast, CD70low tumors were associated with proliferative (p<0.00001) and stromal/proliferative (p=0.005) subtypes. Notably, although CD70high was associated with a trend towards inferior survival in the TCGA, this was not observed in the clinical trial cohorts (compromised of patients with stage IV disease). CD70 expression did not carry a consistent predictive role across these cohorts. Conclusion In RCC, high CD70 expression is strongly associated with the angiogenic subtype. This may serve as rationale for examining combinations of CD70-directed therapies with VEGF inhibitors. Figure 1. NMF cluster by CD70 expression



# Abstract: Associations of abdominal adiposity and skeletal muscle with renal cell carcinoma histological subtype, grade, and stage

**LEAD AUTHOR**

Batai, Ken, Roswell Park Comprehensive Cancer Center

**CO-AUTHORS**

Monica Sarker, University at Buffalo

Juan Adrover Claudio, Roswell Park Comprehensive Cancer Center

Robert M. Blew, University of Arizona

Benjamin R. Lee, University of Arizona

Hina Arif Tiwari, University of Arizona

Patrick Wightman, University of Arizona

Rikki A. Cannioto, Roswell Park Comprehensive Cancer Center

Eric C. Kauffman, Roswell Park Comprehensive Cancer Center

Jennifer W. Bea, University of Arizona

Background: Abdominal adiposity and skeletal muscle influence cancer prognosis, but their associations with renal cell carcinoma pathology remain unclear. We investigated associations between body composition and diagnosis with clear cell histology, high grade, and advanced stage in a diverse cohort.

Methods: Using preoperative CT and MRI scans, we measured area (cm2) and radiodensity (HU; CT scans only) of adipose tissue subcompartments and skeletal muscle at the third lumbar vertebrae level. Area measurements were normalized for height squared (cm^2^/m^2^) to calculate intermuscular, visceral, and subcutaneous adipose tissue indices (IMATI, VATI, SATI) and skeletal muscle index (SMI). Body composition phenotypes were defined by combining sex-specific medians for each adiposity index with SMI (high vs. low). Logistic regression analyses estimated odds ratios (ORs) and 95% confidence intervals (CIs) adjusting for potential confounders. For adipose tissue indices, the second quartile (Q2) was selected as the reference group.

Results: Among 268 patients (56.7% non-Hispanic White, 25.4% Hispanic, 7.1% American Indian, 6.3% non-Hispanic Black), high IMATI (Q4 vs Q2) increased odds of clear cell subtype (OR 5.91, 95% CI:1.55–22.59; P for trend = 0.02), while low IMATI (Q1 vs Q2) reduced odds of advanced stage (OR 0.30, 95% CI:0.12–0.77;

P = 0.01). Each SATI quartile increased odds of high grade (OR per quartile 1.47, 95% CI:0.99–2.19). High VAT radiodensity (Q4 vs Q1) increased odds of advanced stage (OR 4.94, 95% CI:1.24–19.73; P for trend = 0.04). For all adiposity measurements, the low adiposity low SMI phenotype had about three-fold increased odds of high grade compared with the low adiposity high SMI healthy phenotype.

Conclusions: These findings suggest that greater abdominal adiposity and lower skeletal muscle mass is associated with aggressive pathology. Prospective studies are needed to determine if preoperative body composition predicts survival.

# Abstract: Impact of Post-Diagnosis Body Mass Index ( BMI) Change on Survival in Localized Renal Cell Carcinoma

**LEAD AUTHOR**

Sarker, Monica, Roswell Park Comprehensive Cancer Center, University at Buffalo

**CO-AUTHORS**

Eric C. Kauffman, Roswell Park Comprehensive Cancer Center

Li Tang, Roswell Park Comprehensive Cancer Center

Marilyn L. Kwan, Kaiser Permanente Northern California

Zhengyi Deng, Sanford University

Marvin E. Langston, Sanford University

Sumati Gupta University of Utah Huntsman Cancer Institute Huntsman Cancer Institute, G. Daniel Grass

Eric A. Singer, H. Lee Moffitt Cancer Center & Research Institute, The Ohio State University Comprehensive Cancer Center

Yousef Zakharia, University of Iowa Holden Comprehensive Cancer Center

Paul Viscuse, University of Virginia

Laura Graham, University of Colorado Anschultz Medical Campus

Saum Ghodoussipour, Rutgers Cancer Institute of New Jersey

Bodour Salhia, University of Southern California Keck School of Medicine

Sean Kern, Uniformed Services University Murtha Cancer Center and Walter Reed National Military Medical Center

Adanma Ayanambakkan, Oklahoma University Stephensen Cancer Center

Zin Myint, Markey Cancer Center

Jennifer King, Indiana University Simon Cancer Center

Seyi Oderinde, Indiana University Simon Cancer Center

Michelle L. Churchman, Aster Insights

Robert J Rounbehler, Aster Insights

Ken Batai, Roswell Park Comprehensive Cancer Center

Background: Although obesity increases renal cell carcinoma (RCC) risk, obese patients with localized RCC often show better survival, a phenomenon referred to as the “obesity paradox.” However, the effect of post-diagnosis BMI change on prognosis remains unclear. This study aimed to investigate whether post-diagnostic BMI change influences overall survival (OS) and disease-free survival (DFS). Methods:

We analyzed 1414 patients with AJCC stage I–III RCC from the Oncology Research Information Exchange Network (ORIEN) retrospective cohort. All RCC subtypes were included. BMI change was calculated from diagnosis to 2- and 3-years post-diagnosis and categorized as stable (≤5%), 5–10% loss, >10% loss, 5–10% gain, and >10% gain. Multivariable Cox models adjusted covariates and estimated hazard ratios (HRs) for OS and DFS.

Results: At two years post-diagnosis, 16.8% of patients experienced >10% gain, while 10.1% had >10% loss. Compared to patients with a stable BMI, those who lost more than 10% of their BMI had significantly worse OS (HR = 4.87, 95% CI: 2.48–9.57, p < 0.0001) and DFS (HR = 2.12, 95% CI: 1.32–3.39, p = 0.002). Patients with >10% gain at two years showed worse DFS (HR = 1.43, 95% CI: 0.98–2.08, p = 0.07). At three years post-diagnosis, >10% loss remained significantly associated with worse outcomes (OS: HR = 4.87, 95% CI: 2.48–9.57, p < 0.0001; DFS: HR = 2.92, 95% CI: 1.80–4.73, p < 0.0001), and 5–10% BMI loss was also associated with worse DFS (HR = 1.98, 95% CI: 1.19–3.29, p = 0.01).

Conclusion: Post-diagnosis BMI loss was associated with worse survival in stage I–III RCC, suggesting the obesity paradox may partly reflect weight loss from disease progression and highlights the prognostic value of monitoring BMI after diagnosis.

# Abstract: Factors Associated with IVC Thrombectomy for Non-Metastatic, Node-Negative Renal Cell Carcinoma in Octogenarians

**LEAD AUTHOR**

Wong, Vivian, Division of Urologic Oncology, The Ohio State Comprehensive Cancer Center

**CO-AUTHORS**

Rajvi Goradia, Division of Urologic Oncology, The Ohio State Comprehensive Cancer Center

Alyssa Lombardo, Division of Urologic Oncology, The Ohio State Comprehensive Cancer Center

Micheal Bacchus, Division of Urologic Oncology, The Ohio State Comprehensive Cancer Center

Isaac Kim, Department of Urology, Stanford University

Akshay Sood, Division of Urologic Oncology, The Ohio State Comprehensive Cancer Center

Shawn Dason, Division of Urologic Oncology, The Ohio State Comprehensive Cancer Center

Eric Singer, Division of Urologic Oncology, The Ohio State Comprehensive Cancer Center

Comorbidities and performance status are important considerations when counseling patients with non-metastatic renal cell carcinoma (RCC) and IVC tumor thrombus about surgical resection, but it is unclear how patient age impacts surgical selection. Our objective was to evaluate factors associated with IVC thrombectomy for RCC in those aged 80 and older. We hypothesized that patients deemed fit enough for surgery would have an overall survival (OS) advantage over those who were unfit for surgery or declined extirpative therapy.

We examined cT3bN0M0 RCC patients aged 80 and older in the National Cancer Database (NCBD) from 2004–2018. Univariable and multivariable logistic regression models were utilized to evaluate clinical and socioeconomic factors associated with receiving surgery among octogenarians diagnosed with RCC. Kaplan-Meier curves were used to evaluate 5-year OS among octogenarians receiving surgery versus no surgery.

There were 482 octogenarians with cT3bN0M0 RCC. On multivariable analysis, the odds of receiving surgery were lower in octogenarians with increasing age (>84 years) (OR: 0.84 [95%CI: 0.78-0.89], p<0.001) or increasing Charlson-Deyo score >2 (OR=0.44 [95%CI: 0.22-0.87], p=0.02). Patients who lived >29.5 miles from the treating facility had increased odds of undergoing surgery (OR=2.90 [95%CI: 1.71-4.96], p<0.001). Octogenarians who received surgery had higher 5-year OS compared to those without surgery, 83% (95%CI:77-89) vs 70% (95%CI:59-83), p=0.03 [Figure 1].

These data represent the largest study evaluating factors associated with receipt of IVC thrombectomy in octogenarians for cT3bN0M0 RCC. This management dilemma will become increasingly common as more Americans age into their 80s. As expected, increasing age and comorbidity score was associated with lower odds of receiving IVC thrombectomy. However, patients traveling to a medical center had increased likelihood of undergoing surgery and receipt of IVC thrombectomy was associated with a significant OS benefit.



# Abstract: Efficacy and Safety of Bevacizumab plus Everolimus in Pretreated Metastatic non-clear cell RCC

**LEAD AUTHOR**

Ocejo Gallegos, Jesus Antonio, Department of Medicine, Memorial Sloan Kettering Cancer Center, New York, NY

**CO-AUTHORS**

Andrea Knezevic, Department of Epidemiology and Biostatistics, Memorial Sloan Kettering Cancer Center, New York, NY

Kira Vom Eigen, Department of Medicine, Memorial Sloan Kettering Cancer Center, New York, NY

Maria Isabel Carlo, Department of Medicine, Memorial Sloan Kettering Cancer Center, New York, NY; Department of Medicine, Weill Cornell Medical College, New York, NY

Andrew Cornish, Department of Medicine, Memorial Sloan Kettering Cancer Center, New York, NY

Sahil D. Doshi, Department of Medicine, Memorial Sloan Kettering Cancer Center, New York, NY

Ritesh Kotecha, Department of Medicine, Memorial Sloan Kettering Cancer Center, New York, NY; Department of Medicine, Weill Cornell Medical College, New York, NY

Neil J. Shah, Department of Medicine, Memorial Sloan Kettering Cancer Center, New York, NY; Department of Medicine, Weill Cornell Medical College, New York, NY

Robert J. Motzer, Department of Medicine, Memorial Sloan Kettering Cancer Center, New York, NY; Department of Medicine, Weill Cornell Medical College, New York, NY

Darren R. Feldman, Department of Medicine, Memorial Sloan Kettering Cancer Center, New York, NY; Department of Medicine, Weill Cornell Medical College, New York, NY

Martin H. Voss, Department of Medicine, Memorial Sloan Kettering Cancer Center, New York, NY; Department of Medicine, Weill Cornell Medical College, New York, NY

Background: In a prior phase 2 study we confirmed efficacy and safety of bevacizumab plus everolimus in untreated metastatic non-clear cell renal cell carcinoma (ncRCC), which led to NCCN compendium listing in this space. Here, we report on our group’s experience with this regimen in systemically pre-treated ncRCC patients, particularly following immune checkpoint inhibitor (ICI) exposure. Methods: We conducted retrospective chart review of patients with metastatic ncRCC treated with bevacizumab/everolimus as second or later line therapy. Using Kaplan-Meier method, we determined time to treatment failure (TTF), defined as time from treatment start to permanent discontinuation for progression, toxicity or death, as well as overall survival (OS).

Results: Twenty-seven patients were included: papillary (n=12, 44%), unclassified (n=7, 26%), chromophobe (n=5, 19%), translocation (n=2, 7%), and FH-deficient (n=1, 4%) RCC. The median number of prior treatment lines was 1 (range 1–7); 85% (n=23) had received prior ICI therapy, and 89% (n=24) underwent nephrectomy. All patients discontinued bevacizumab/everolimus: 81% (n=22) due to progression and 19% (n=5) due to toxicity (pneumonitis 3, proteinuria 1, hematoma 1). Median TTF was 4.1 months (95% CI: 1.9-5.4). Six patients (22%) discontinued at the time of the first scan, while three patients (11%) remained on therapy for >12 months. Among surviving patients (n=2), follow-up was 9 and 34 months. Twelve-month OS was 36% (95% CI: 19, 54).

Conclusion: In this real-world cohort, bevacizumab/everolimus demonstrated limited efficacy in IO-pretreated ncRCC with manageable toxicity. A small subset of patients experienced durable clinical benefit, though no predictive clinical or histopathologic features could be identified.



# Abstract: Local recurrence after nephron-sparing treatment of renal cell carcinoma – a risk factor for metastases and early death

**LEAD AUTHOR**

Ljungberg, Borje, Department of Diagnostics and Intervention, Urology and Andrology, Umeå University, Umeå, Sweden

**CO-AUTHORS**

Bassam Mazin Hashime, Center for clinical research, county of Västmanland, Department of Surgical Sciences, Uppsala University, Uppsala, Sweden

Per Lindblad, School of Medical Sciences, Faculty of Medicine and Health, Örebro University, Örebro, Sweden

Andreas Karlsson Rosenblad, Regional Cancer Centre Stockholm-Gotland, Region Stockholm, Stockholm, Sweden, Department of Statistics Uppsala University, Uppsala, and Department of Medical Sciences, Division of Clinical Diabetology and Metabolism, Uppsala University, Uppsala, Sweden

Objective: To explore the risks for distant metastases and death after renal cell carcinoma (RCC) local recurrence in nephron-sparing treated patients using ablation therapy (AT) or partial nephrectomy (PN). Furthermore, to evaluate overall mortality in patients without disease recurrence.

Methods: Nation-wide population-based data on 2701 patients (2761 individual tumors) with nonmetastatic RCC, AT and PN treated during 2005-2018, were extracted from the National Swedish Kidney Cancer Register. Time to local tumor recurrence or death, with or without local recurrence, was analysed using Cox regression models.

Results: After a mean follow-up of 4.8 years, local recurrence was observed for 111 (4.0%) tumours, and death without any disease recurrence for 206 (7.5%) patients. Based on the offered primary treatment, AT-treated tumours had a 4.3 times higher risk of local recurrence (P < 0.001) than PN-treated. After 3.2 years mean follow-up after the occurrence of the local recurrence, 24 (22%) of these patients had died, compared with 7.5% death in patients without any disease recurrence. There were no significant differences in overall survival between AT- and PN-treated patients having no sign of disease recurrence.

Conclusions: Local RCC recurrence after nephron-sparing treatment implicates a significantly increased risk for death. Patients treated with AT had a higher risk of local recurrence compared with PN. Since there were no significant differences regarding risk of death between AT- and PN-treated patients without any disease recurrence, the major goal is to achieve a careful technical AT as well as a careful PN, to reduce all risks of leaving any tumor cells behind at the primary RCC treatment.

# Abstract: Effect of Prior Solid Organ Transplant on Localized RCC Outcomes by Treatment Type

**LEAD AUTHOR**

Roadman, Daniel, University of Wisconsin School of Medicine and Public Health

**CO-AUTHORS**

E. Jason Abel, University of Wisconsin School of Medicine and Public Health

Parth Shah, University of Wisconsin School of Medicine and Public Health

Andrew Z. Carey, University of Wisconsin School of Medicine and Public Health

Paz Lotan, University of Wisconsin School of Medicine and Public Health

Joshua Mezrich, University of Wisconsin School of Medicine and Public Health

David Al-Adra, University of Wisconsin School of Medicine and Public Health

David Aufhauser, University of Wisconsin School of Medicine and Public Health

Kelly M. Collins, University of Wisconsin School of Medicine and Public Health

Didier Mandelbrot, University of Wisconsin School of Medicine and Public Health

Sandesh Parajuli, University of Wisconsin School of Medicine and Public Health

Glenn O. Allen, University of Wisconsin School of Medicine and Public Health

David F. Jarrard, University of Wisconsin School of Medicine and Public Health

Kyle A. Richards, University of Wisconsin School of Medicine and Public Health

Elizabeth Koehne, University of Wisconsin School of Medicine and Public Health

Michael Risk, University of Wisconsin School of Medicine and Public Health

Daniel D. Shapiro, University of Wisconsin School of Medicine and Public Health

Introduction: Renal cell carcinoma (RCC) in solid organ transplant recipients poses unique challenges due to immunosuppression, which increases both cancer risk and non-cancer mortality. Evidence guiding treatment—including surgery, ablation, and active surveillance—is limited, and current guidelines lack robust data. This study aimed to evaluate the impact of transplant status on oncologic outcomes across different RCC treatment strategies compared to a non-transplant cohort.

Methods: We identified 81 transplant recipients with cT1 RCC and matched them 1:2 to 162 non-transplant controls by age, tumor size, and tumor grade. Patients were categorized by treatment: surgery, ablation, or active surveillance. Baseline characteristics and survival outcomes were compared between transplant and non-transplant cohorts. Cox proportional hazard models evaluated associations between transplant status and survival, adjusting for confounders.

Results: Among transplant recipients (n=81), median age was 57 years, with clear cell (43%) and papillary (41%) as the most common histologies. Initial treatments included surgery (65%), ablation (23%), and active surveillance (10%). Median tumor size was 3.0 cm, and median follow-up was 68 months. The 3-year MFS and OS were 97% and 88%, respectively.

Among surgical patients (non-transplant n=104, transplant n=53), transplant patients had higher comorbidities, and postoperative complications (11% vs. 3%, P=0.04). MFS and OS were similar (Fig 1A). In the ablation cohort (non-transplant n=43, transplant n=19), transplant patients had more papillary RCC (58% vs 19%, P=0.005). Again, there was no difference in MFS or OS compared to non-transplant patients (Fig 1B). In the surveillance cohort (non-transplant n=16, transplant n=9), no significant differences were found in MFS or OS (Fig 1C).

Conclusions: Patients with cT1 RCC and prior transplantation experienced similar oncologic outcomes as non-transplant controls across all treatment modalities. These findings support the safe use of surgery, ablation, and surveillance in appropriately selected transplant recipients, highlighting the need for prospective validation.



# Abstract: KEYMAKER-U03 substudy 03C: Phase 1b/2 study of belzutifan plus zanzalintinib for recurrent clear cell renal cell carcinoma (RCC) during or after anti–PD-(L)1 therapy

**LEAD AUTHOR**

Rini, Brian, Vanderbilt-Ingram Cancer Center, Nashville, OH, USA

**CO-AUTHORS**

Laurence Albiges, Gustave Roussy, Université Paris-Saclay, Villejuif, France

Cristina Suárez, Vall d´Hebron Institute of Oncology (VHIO), Hospital Universitari Vall d ´Hebron, Vall d´Hebron Barcelona Hospital Campus, Barcelona, Spain

Thomas Powles, Barts Health Biomedical Research Center, Queen Mary’s University of London ECMC, UK

Victoria Neiman, Davidoff Cancer Center, Rabin Medical Center, Petah Tikva, Israel Brian Simmons, Exelixis, Inc., Alameda, CA, USA

Lockman Bousserouel, Merck & Co., Inc., Rahway, NJ, USA

Joseph E. Burgents, Merck & Co., Inc., Rahway, NJ, USA

Manish Sharma, Merck & Co., Inc., Rahway, NJ, USA

Robert J Motzer, Memorial Sloan Kettering Cancer Center, New York, NY, USA

Background: Adjuvant pembrolizumab is a standard of care for patients with RCC at intermediate-high or high risk of recurrence postsurgery. Recurrence after adjuvant anti–PD-(L)1 therapy requires a novel treatment approach. Substudy 03C of the open-label, phase 1b/2 KEYMAKER-U03 trial (NCT07049926) is designed to evaluate the safety and efficacy of belzutifan plus zanzalintinib for recurrent RCC during or after anti–PD-(L)1 adjuvant therapy.

Methods: Eligible participants (≥18 years) must have histologically confirmed unresectable locally advanced or metastatic clear cell RCC, measurable disease per RECIST v1.1 as assessed by investigator and verified by blinded independent central review (BICR), and KPS ≥70%. The study will comprise a safety lead-in phase followed by an efficacy phase. In the safety-lead in, prior immunotherapy and ≤1 VEGF-TKI for metastatic disease is permitted. Participants in the efficacy phase must not have received prior systemic treatment except for adjuvant anti–PD-(L)1 therapy and had recurrence during or ≤24 months after the last dose of adjuvant anti–PD-(L)1 therapy. Treatment will consist of belzutifan plus zanzalintinib (arm C1). Additional treatment arms may be added on a rolling basis. In the safety lead-in phase, ≥10 participants will receive belzutifan 120 mg orally QD plus zanzalintinib 60 mg (arm C1a) or 100 mg (arm C1b) orally QD. The primary objective of the safety lead-in phase is to establish the recommended phase 2 dose (RP2D). In the efficacy phase, approximately 80 participants will receive belzutifan plus zanzalintinib at the RP2D. Co-primary end points of the efficacy phase are safety and ORR per RECIST v1.1 by BICR. Secondary end points are CBR, DOR and PFS per RECIST v1.1 by BICR, and OS. This study is currently enrolling.

# Abstract: Strategic Trimodal Therapy Enhances Radiation-induced Abscopal Response in Renal Cancer

**LEAD AUTHOR**

Pal, Krishnendu, Department of Biochemistry and Molecular Biology, Mayo Clinic College of Medicine and Science, Jacksonville, Florida

**CO-AUTHORS**

Hari Krishnareddy Rachamala, Department of Biochemistry and Molecular Biology, Mayo Clinic College of Medicine and Science, Jacksonville, Florida

Naga Malleswara Rao Nakka, Department of Biochemistry and Molecular Biology, Mayo Clinic College of Medicine and Science, Jacksonville, Florida

Shamit Kumar Dutta, Department of Biochemistry and Molecular Biology, Mayo Clinic College of Medicine and Science, Jacksonville, Florida

Enfeng Wang, Department of Biochemistry and Molecular Biology, Mayo Clinic College of Medicine and Science, Jacksonville, Florida

Debabrata Mukhopadhyay, Department of Biochemistry and Molecular Biology, Mayo Clinic College of Medicine and Science, Jacksonville, Florida

Abscopal effect is typically observed in sporadic cases even in immunogenic cancers like renal cancer. Despite various attempts, no effective strategy has been developed to enhance this effect. We developed a novel approach combining a tumor-targeted liposomal formulation of everolimus and YM155 (EY-L) with radiation and interleukin-2 (IL-2) to boost this effect. Using two murine renal cancer models, we tested this trimodal therapy in a bilateral tumor setup to assess local and distant tumor responses. The combination of EY-L, radiation, and IL-2 led to marked suppression of both irradiated and non-irradiated tumors in the Renca model, indicating a robust enhancement of the abscopal effect. Importantly, removal of any of the components of this trimodal therapy failed to produce a similar response, emphasizing the necessity of all three components. Mechanistically, this effect correlated with increased CD8+ T cell infiltration and concomitant reduction in CD163+ M2-like macrophage population in non-irradiated tumors, suggesting enhanced systemic anti-tumor immunity. In the LVRCC67 model, this trimodal therapy enhanced the abscopal response; however, the effect was somewhat smaller, likely due to the model’s immune-excluded nature. Nonetheless, these findings reveal a promising strategy to reliably induce the abscopal effect through a synergistic therapeutic combination, offering a path toward more effective immunoradiotherapy regimens. In summary, our study introduces a targeted, immune-modulatory platform with strong translational potential for improving outcomes in renal and possibly other cancers where the abscopal effect remains elusive.

# Abstract: Outcomes of Patients with Fumarate Hydratase – Deficient Renal Cell Carcinoma Treated with Cabozantinib + Nivolumab

**LEAD AUTHOR**

Carlo, Maria, MSKCC

**CO-AUTHORS**

Ava Jahn, MSKCC

Ying-bei Chen, MSKCC

Sahil Doshi, MSKCC

Andrew Cornish, MSKCC

Ziad El Bakouny, MSKCC

Neil Shah, MSKCC

A Ari Hakimi, MSKCC

Robert Motzer, MSKCC

Martin Voss, MSKCC

Ritesh Kotecha, MSKCC

Darren Feldman, MSKCC

Purpose: Fumarate hydratase-deficient renal cell carcinoma (FH-RCC) is a rare and aggressive type of kidney cancer without a standard form of treatment. It predominantly occurs in individuals with hereditary leiomyomatosis and RCC syndrome (HLRCC), characterized by a germline heterozygous variant in the FH gene. The objective of this study was to evaluate the efficacy of the combination of cabozantinib plus nivolumab in this patient population.

Methods: We identified patients with FH-RCC at our institution based on pathologic diagnosis and/or germline or somatic FH mutations reported on our institutional next-generations sequencing platform MSK-IMPACT. Patient and tumor characteristics, along with length of time on treatment, were recorded. The endpoint of efficacy was measured by the duration, in months, on cabozantinib + nivolumab combination therapy. We used the Kaplan-Meier estimate for median time on treatment.

Results: We identified 36 FH-RCC patients, of which 14 received cabozantinib and nivolumab. Of these, the median age at diagnosis was 38; range 23-73; M:F, 11:3). Of these, 12 (86%) tested positive for a pathogenic germline FH variant and 2 were sporadic; only 2 patients had a known family history of RCC. All evaluable tumors were high grade at initial RCC diagnosis. Most patients (93%) received cabozantinib + nivolumab as first or second line treatment, with 1 patient receiving as fifth line. Median treatment duration was 24.3 months (95% Confidence Interval 21.2 – 35.8 months). Among these, 3 patients (21%) remained on therapy for ≥30 months (Figure 1.). Of 2 patients that received bevacizumab + erlotinib prior to cabozantinib and nivolumab, time on cabozantinib and nivolumab was 21.0 and 30.5 months.

Conclusion: Based on the available data, cabozantinib + nivolumab combination therapy shows promising results as a treatment option for FH-RCC patients.



# Abstract: Nivolumab plus ipilimumab (NIVO+IPI) vs sunitinib (SUN) for first-line treatment of advanced renal cell carcinoma (aRCC): final analysis from the phase 3 CheckMate 214 trial

**LEAD AUTHOR**

George, Saby, Roswell Park Cancer Institute

**CO-AUTHORS**

Toni K. Choueiri, Lank Center for Genitourinary Oncology, Dana-Farber Cancer Institute, Brigham and Women *’*s Hospital, and Harvard Medical School

Bernard Escudier, Gustave Roussy

David F. McDermott, Beth Israel Deaconess Medical Center, Dana-Farber/Harvard Cancer Center

Mauricio Burotto, Bradford Hill Clinical Research Center

Hans J. Hammers, UT Southwestern Kidney Cancer Program

Elizabeth R. Plimack, Fox Chase Cancer Center

Camillo Porta, University of Pavia

Thomas Powles, Barts Cancer Institute, Cancer Research UK Experimental Cancer Medicine Centre, Queen Mary University of London, Royal Free National Health Service Trust

Frede Donskov, University Hospital, Aarhus, Denmark; and Southern Denmark University Hospital

Michael B. Atkins, Georgetown Lombardi Comprehensive Cancer Center

Christian K. Kollmannsberger, British Columbia Cancer Agency

Marc-Oliver Grimm, Jena University Hospital Yoshihiko Tomita, Niigata University Graduate School of Medical and Dental Sciences

Brian I. Rini, Vanderbilt University Medical Center

Ruiyun Jiang, Bristol Myers Squibb

Maximiliano van Kooten Losio, Bristol Myers Squibb

Chung-Wei Lee, Bristol Myers Squibb

Nizar M. Tannir, University of Texas MD Anderson Cancer Center

Robert J. Motzer, Memorial Sloan Kettering Cancer Center

Background: First-line NIVO+IPI provided substantial long-term survival benefits over SUN in patients with aRCC in CheckMate 214.

Methods: Patients with clear cell aRCC were randomized 1:1 to NIVO 3 mg/kg + IPI 1 mg/kg Q3W×4 then NIVO (3 mg/kg or 240 mg Q2W or 480 mg Q4W); or SUN 50 mg once daily (4 weeks on, 2 weeks off). Efficacy endpoints: overall survival (OS), and independent radiology review committee-assessed progression-free survival (PFS) and objective response rate in IMDC intermediate/poor-risk (primary), intent-to-treat (secondary), and favorable-risk (exploratory) patients. Post-hoc analyses of OS were conducted in all patients treated with NIVO+IPI with an immune-mediated adverse event (IMAE) leading to discontinuation.

Results: With 9 years median follow-up, the OS hazard ratio (HR) with NIVO+IPI vs SUN was 0.71 in intent-to-treat and 0.69 in intermediate/poor-risk patients; 108-month OS probabilities favored NIVO+IPI (Table). In favorable-risk patients, the OS HR improved from 1.45 (Motzer NEJM 2018) to 0.80 over 9 years, showing a delayed benefit with NIVO+IPI; 108-month OS probabilities were 35% vs 22% (Table). At 96 months, PFS probabilities were consistent with previous reports and duration of response was longer with NIVO+IPI vs SUN regardless of IMDC risk (Table). No new treatment-related deaths occurred. In post-hoc analyses of 103 patients who discontinued NIVO+IPI due to IMAEs, the 108-month OS probability was 40%. For intent-to-treat patients, the 108-month OS probability was 31%. Additional post-hoc efficacy analyses of patients who discontinued NIVO+IPI due to IMAEs will be presented.

Conclusions: In the longest and final phase 3 follow-up of a first-line checkpoint inhibitor combination in aRCC, improved survival and durable responses were maintained with NIVO+IPI over SUN. No new safety signals emerged.

Copyright: This is an adapted abstract. “Nivolumab plus ipilimumab vs sunitinib for first-line treatment of advanced renal cell carcinoma: final analysis from the phase 3 CheckMate 214 trial” was previously presented at the 2025 American Society of Clinical Oncology (ASCO) Annual Meeting; May 30-June 3; Chicago, IL.

**Nivolumab plus ipilimumab (NIVO+IPI) vs sunitinib (SUN) for first-line treatment of advanced renal cell carcinoma (aRCC): final analysis from the phase 3 CheckMate 214 trial**

**Table:** Table

	Intent-to-treat		Intermediate/poor risk		Favorable risk	
**Arm; n**	**NIVO+IPI; 550**	**SUN; 546**	**NIVO+IPI; 425**	**SUN; 422**	**NIVO+IPI; 125**	**SUN; 124**
**mOS (95% CI), mo**	53 (46–64)	38 (32–44)	47 (35–56)	26 (22–33)	78 (65–92)	67 (56–80)
**108-mo OS probabilities**	31 (27–35)	20 (16–23)	30 (26–35)	19 (15–23)	35 (27–44)	22 (15–30)
**mPFS (95% CI), moa**	12 (10–17)	12 (10–15)	12 (9–17)	9 (7–11)	13 (10–18)	29 (23–43)
**96-mob PFS probabilities**	23 (18–27)	9 (5–15)	25 (20–31)	9 (4–15)	13 (6–22)	11 (3–27)
**ORR per IRRC (95% CI); CR, %a**	39 (35–44); 12	33 (29–37); 3	42 (38–47); 12	27 (23–32); 3	30 (22–38); 13	52 (43–61); 6
**mDOR (95% CI), moa**	76 (59–NE)	25 (20–33)	83 (54–NE)	20 (16–26)	61 (23–NE)	33 (25–51)
**96-mob DOR probabilitie (95% CI), %a**	48 (39–55)	19 (10–31)	50 (41–58)	23 (13–36)	36 (17–56)	NAc

^a^Response assessed using RECIST v1.1. b96-month probabilities reported due to small numbers of patients at risk at 108 months. cNo patients remain at risk. CR, complete response; DOR, duration of response; IRRC, independent radiology review committee; m, median; NA, not applicable; NE, not estimable; ORR, objective response rate.

# Abstract: Exploring the impact of combined anti-angiogenic and immune checkpoint inhibitors in clear cell renal cell carcinoma metastases

**LEAD AUTHOR**

Dondossola, Eleonora, UT MD Anderson Cancer Center

**CO-AUTHORS**

Stefan Maksimovic, UT MD Anderson Cancer Center

Gerardo Murgolo, UT MD Anderson Cancer Center

Matthew Campbell, UT MD Anderson Cancer Center

Clear cell renal cell carcinoma (ccRCC) is the most common kidney cancer, frequently spreading to bone and lungs. A hallmark of ccRCC is loss of von Hippel– Lindau (VHL) protein, promoting angiogenesis; in addition, ccRCC is characterized by a complex and heterogenous immune infiltrate. This has led to the use of anti-angiogenic tyrosine kinase inhibitors (TKIs) and immune checkpoint inhibitors (ICIs), alone or in combination, as standard of care for ccRCC patients. However, their site-specific efficacy and effects on the immune microenvironment remain unclear. We hypothesized that TKIs modulate the tumor microenvironment to enhance ICI efficacy, with outcomes influenced by metastatic location, drug dose, and tumor size.

To test this, we implanted a luciferase-GFP-labeled RENCA-VHL- cell line into the tibia of immunocompetent mice, inducing synchronous bone and lung metastases. Mice were treated with cabozantinib (cabo), anti-PD1, and anti-CTLA4 antibodies, either alone or combined. Tumor progression was assessed via in vivo bioluminescence and ex vivo 3D spatial analysis.

All treatments reduced tumor burden at both sites compared to controls. Post-treatment withdrawal revealed efficacy differences. Spatial profiling showed treatment- and site-specific changes in tumor cells, immune infiltrates, and vasculature, with potential TKI-ICI synergy.

Our immunocompetent mouse model confirms the efficacy of current therapies in bone and lung metastases and highlights key differences in microenvironmental response, providing important insights and efficacy predictions for therapeutic applications in metastatic ccRCC patients.

# Abstract: Preliminary Phase 1 safety and antitumor activity of XmAb819, a first-in-class ENPP3 x CD3 bispecific antibody, in patients with advanced clear cell renal cell carcinoma (ccRCC)

**LEAD AUTHOR**

Kumar Pal, Sumanta, City of Hope

**CO-AUTHORS**

Randy F Sweis, University of Chicago Medicine

Ritesh R Kotecha, Memorial Sloan Kettering Cancer Center

Karie Runcie, Columbia University Mehmet Bilen, Winship Cancer Institute at Emory University

Pavlos Msaouel, The University of Texas MD Anderson Cancer Center

David A Braun, Yale Cancer Center

Scott S Tykodi, University of Washington and Fred Hutchinson Cancer Center

Christopher Hoimes, Duke University Medical Center

Chet Bohac, Xencor, Inc.

Vithika Suri, Xencor, Inc.

Jitendra Kanodia, Xencor, Inc.

Engie Salama, Xencor, Inc.

Jacky Woo, Xencor, Inc.

Selam Berhe, Xencor, Inc.

Yuanquan Yang, The Ohio State University Wexner Medical Center

Robert Franklin, University of Cincinnati Medical Center

Claud Grigg, Atrium Health

Joseph Maly, Norton Cancer Center

Shuchi Gulati, University of California Davis Comprehensive Cancer Center

David J VanderWeele, Northwestern University

Adanma Ayanambakkam, University of Oklahoma Health Sciences Center

Biren Saraiya, Rutgers Cancer Institute of NJ

Background: XmAb819 is a T-cell engaging bispecific antibody in development for patients with ccRCC. Despite advances in the treatment of metastatic ccRCC, few patients are cured, and therapies exploiting novel targets are needed. Antigen screening identified ectonucleotide pyrophosphatase/phosphodiesterase family member 3 (ENPP3) as having consistent high expression in ccRCC and low expression in normal tissue. XmAb819 utilizes a multivalent 2+1 format with high-avidity bivalent binding to ENPP3 and low-affinity monovalent binding to CD3, a component of the T-cell receptor (TCR) complex. XmAb819 is engineered for preferential engagement of high ENPP3-expressing cancer cells to induce T-cell-mediated cytolysis.

Methods: XmAb819-01 is a Phase 1, multicenter, dose-escalation and expansion study for patients with advanced ccRCC. Patients with previously treated relapsed or refractory ccRCC were enrolled at escalating doses of XmAb819 administered intravenously (IV). XmAb819 was administered QW until disease progression or unacceptable toxicity. Adverse events were graded using CTCAE v5.0 and cytokine release syndrome (CRS) using ASTCT Consensus Grading. Efficacy was assessed by investigator using RECIST v1.1.

Results: As of June 30, 2025, 48 patients with ccRCC were enrolled in 9 cohorts; median age was 60 years, with a median of 4 prior lines of therapy (range, 1-8). The most common treatment-related adverse events (TRAEs) (≥15% any grade) were rash (90%), CRS (90%), fatigue (46%), diarrhea (35%), vomiting (31%), nausea (27%), increased aminotransferase levels (21%), pruritus (19%), chills (17%), decreased lymphocytes (17%), and pyrexia (17%). The most common (≥10%) Grade ≥3 TRAEs were decreased lymphocytes (17%), CRS (15%), and rash (13%). Rash was transient and resulted in no discontinuations. CRS most commonly followed the first dose of treatment. One dose-limiting toxicity of Grade 4 elevated liver enzymes was deemed related to treatment. No cases of treatment-related immune effector cell-associated neurotoxicity syndrome (ICANS) were observed. No treatment-related Grade 5 events were observed. Eight patients (17%) remain on treatment; 40 patients (83%) discontinued treatment, 27 (56%) due to progressive disease (PD), 4 (8%) due to clinical progression, 4 (8%) due to adverse event (increased aminotransferase levels, myocardial infarction, elevated liver enzymes, and hematuria; 1 patient each), 2 (4%) due to the patient withdrawal, and 1 (2%) each due to investigator’s decision, death (due to PD), or intolerance to treatment. Preliminary evidence of antitumor activity has been observed, including confirmed partial responses.

Conclusions: XmAb819 is safe and well-tolerated at IV doses that induce antitumor activity in patients with advanced ccRCC. A recommended Phase 2 dose has not yet been determined. IV dose escalation continues.

# Abstract: Cabozantinib in patients (pts) with nonlocally pretreated brain metastases (BM) from renal cell carcinoma (RCC) : Results from the multicenter CABRAMET phase II trial

**LEAD AUTHOR**

NEGRIER, Sylvie, Centre Léon Bérard, Lyon, University Lyon I, FRANCE

**CO-AUTHORS**

Loïc Mourey, Oncopole Claudius Regaud, University Institute of Cancer, IUCT Toulouse-Oncopole, France

Cecile DALBAN, Centre Léon Bérard, Lyon, FRANCE

Marine Gross-Goupil, Department of Medical Oncology, Bordeaux University Hospital, Bordeaux, France

Aude FLECHON, Centre Léon Bérard, Lyon, FRANCE

Bernard Escudier, Institut Gustave Roussy, Villejuif, FRANCE

Philippe Barthélémy, CHU Strasbourg, FRANCE

Stéphane Oudard, Hopital G Pompidou, AP-HP, Université Paris-Cité, Paris, FRANCE

Emeline Colomba, Institut Gustave Roussy, Villejuif, FRANCE

Constance Thibault, Hopital G Pompidou, AP-HP, Paris, FRANCE

Alain RAVAUD, Department of Medical Oncology, Bordeaux University Hospital, Bordeaux, France

Christine Chevreau, Oncopole Claudius Regaud, University Institute of Cancer, IUCT Toulouse-Oncopole, France

Helen Boyle, Centre Léon Bérard, Lyon, FRANCE

Ellen Blanc, Centre Léon Bérard, Lyon, FRANCE

Sylvie Chabaud, Centre Léon Bérard, Lyon, FRANCE

David Pérol, Centre Léon Bérard, Lyon, FRANCE

Laurence Albiges, Institut Gustave Roussy, Université Paris-Saclay, Villejuif, FRANCE

Ronan Flippot, Institut Gustave Roussy, Villejuif, FRANCE

Background

Brain metastases of renal cell cancer are observed in 10 to 15% of patients, but the effect of medical treatments was reported in 3 previous prospective trials only. The phase II trial CABRAMET evaluates the efficacy of the VEGFR tyrosine kinase inhibitor (TKI) cabozantinib in patients with non-locally pretreated brain metastases (BrM) from renal cell carcinoma (RCC).

Patients and Methods

This multicenter open-label trial included RCC patients with BrM with less than three prior systemic treatments excluding cabozantinib. Eligible patients received 60mg/day oral cabozantinib with dose adjustments for toxicity. The primary endpoint was the 6-month progression-free rate in brain metastases (6m-BrMPFR). The main secondary endpoints included BrM objective response rate (ORR) and response duration in BrM, extra-cranial ORR, BrM and overall progression-free survival (PFS), overall survival (OS), and safety.

Results

The study enrolled 26 patients, with a median follow-up of 38.8 months (range 28.3-52.7 months). The 6m-BrM-PFR was 56% (unilateral 95%CI 37.9-) by central review. BrM partial responses were achieved in 16/26 patients (BrM ORR of 61.5%) by investigator assessment. The median BrM response duration was not reached and 58.3% (95%CI 29.3–78.9%) of the patients were event-free at 24 months. The median BrM-PFS was 10.7 (95%CI 5.4-NR) months, and the median overall PFS was 8.1 (95%CI 4-11.9) months. The median OS was 15.0 (95%CI 9.3– 35.0) months. Cabozantinib exhibited significant efficacy as first-line treatment with BrM ORR of 86%, 67% in patients with prior immunotherapy, 40% in patients previously treated with TKI.

Conclusion

The CABRAMET trial is the first to prospectively assess cabozantinib in non-locally pretreated brain metastases from RCC, highlighting its prolonged efficacy and tolerability. These results demonstrate the promising use of cabozantinib in treating this challenging patient population, and may represent a new option in the treatment strategy.

# Abstract: A phase II study of neoadjuvant ivonescimab in patients with high-risk, localized RCC

**LEAD AUTHOR**

Yip, Wesley, City of Hope

**CO-AUTHORS**

Salvador Jaime Casas, City of Hope

Clayton Lau, City of Hope

Ali Zhumkhawala, City of Hope

Jonathan Yamzon, City of Hope

Bertram Yuh, City of Hope

Alexander Chehrazi Raffle, City of Hope

Abhishek Tripathi, City of Hope

Tanya Dorff, City of Hope

Sumanta Pal, City of Hope

Background:

Approximately 70% of renal masses present as localized disease, but there remains a significant concern for relapse in patients with high-risk disease treated by surgery alone. Prior neoadjuvant studies of single agent immune checkpoint or tyrosine kinase inhibition have demonstrated low rates of radiographic or pathologic response. Ivonescimab, a first-in-class anti-PD1/VEGF bispecific antibody, has recently shown superior clinical activity over pembrolizumab in non-small cell lung cancer. Co-targeting VEGF and PD-1 with combination regimens has been successful in metastatic RCC and demonstrated promise in the neoadjuvant setting, as the majority of cases are driven by somatic alterations in the VHL gene, which in turn leads to upregulation of VEGF. However, a bispecific approach has not yet been evaluated. Thus, we have developed the following clinical trial of neoadjuvant ivonescimab in patients with high-risk, localized RCC.

Methods:

This is an open-label, non-randomized, preoperative Phase II study of ivonescimab in the neoadjuvant setting for the treatment of patients with high-risk, localized RCC planned for nephrectomy. Potentially eligible patients will be consented and undergo a baseline biopsy to establish histologic confirmation of clear cell RCC. Ivonescimab will be administered at a dose of 20 mg/kg IV, Q3W administered for 60 minutes (± 10 minutes). After 4 cycles (12 weeks) of study treatment, surgical resection will follow. Surgery will be scheduled at least 4 weeks after the last cycle of ivonescimab. The primary endpoint of this trial is objective response (either CR or PR) by RECIST 1.1 prior to surgery. Secondary endpoints include toxicity, safety, and recurrence-free survival. Exploratory endpoints include KIM-1 levels, genomic signature expression, and quality of life assessments by FKSI-23.



# Abstract: Dietary fiber intake and gut microbial fiber fermentation capacity are associated with favorable clinical outcomes with immune checkpoint blockade (ICB) for renal cell carcinoma (RCC)

**LEAD AUTHOR**

Guercio, Brendan, Wilmot Cancer Institute, University of Rochester Medical Center, Rochester, NY, USA

**CO-AUTHORS**

Joshua Fein, Division of Hematology & Medical Oncology, Meyer Cancer Center, Weill Cornell Medicine, New York, NY, USA

Anqi Dai, Adult Bone Marrow Transplantation Service, Department of Medicine, Memorial Sloan Kettering Cancer Center, New York, NY, USA

Neha Ratna, Genitourinary Oncology Service, Department of Medicine, Memorial Sloan Kettering Cancer Center, New York, NY, USA

Ebrahimi Hedyeh, Beth Israel Deaconess Medical Center, Boston, MA, USA

Keehoon Lee, Translational Genomics Research Institute An Affiliate of City of Hope, Flagstaff, AZ, USA

Lauren Harford, Adult Bone Marrow Transplantation Service, Department of Medicine, Memorial Sloan Kettering Cancer Center, New York, NY, USA

Nicholina Metzker, Genitourinary Oncology Service, Department of Medicine, Memorial Sloan Kettering Cancer Center, New York, NY, USA

Arielle Elkrief, Centre de recherche du CHUM, Montreal, Canada

Zoe Katsamakis, Adult Bone Marrow Transplantation Service, Department of Medicine, Memorial Sloan Kettering Cancer Center, New York, NY, USA

Christopher Ford, On behalf of the SER-155 Team at Seres Therapeutics, Inc., Cambridge, MA, USA

Joshua Drago, Breast Medicine Service, Department of Medicine, Memorial Sloan Kettering Cancer Center, New York, NY, USA

Adam Schoenfeld, Thoracic Oncology Service, Department of Medicine, Memorial Sloan Kettering Cancer Center, New York, NY, USA

Martin Voss, Genitourinary Oncology Service, Department of Medicine, Memorial Sloan Kettering Cancer Center, New York, NY, USA

Robert Motzer, Genitourinary Oncology Service, Department of Medicine, Memorial Sloan Kettering Cancer Center, New York, NY, USA

Jonathan Rosenberg, Genitourinary Oncology Service, Department of Medicine, Memorial Sloan Kettering Cancer Center, New York, NY, USA

Sumanta Pal, Department of Medical Oncology, City of Hope Comprehensive Cancer Center, Duarte, CA

Gopa Iyer, Genitourinary Oncology Service, Department of Medicine, Memorial Sloan Kettering Cancer Center, New York, NY, USA

Ritesh Kotecha, Genitourinary Oncology Service, Department of Medicine, Memorial Sloan Kettering Cancer Center, New York, NY, USA

Jonathan Peled, Adult Bone Marrow Transplantation Service, Department of Medicine, Memorial Sloan Kettering Cancer Center, New York, NY, USA

Gut microbiome composition is associated with ICB efficacy and is influenced by diet. However, mechanisms underlying relationships between the microbiome, diet and ICB remain poorly defined. Butyrate is an immunomodulatory product of microbial fiber fermentation. In a prospective study of 48 evaluable patients with advanced RCC initiating ICB-based therapy, we identified an association between dietary fiber intake ≥15 g/day (average intake in the United States) and longer progression-free-survival (PFS; log-rank p=0.005) and overall survival (OS; log-rank p<0.001; median follow-up 17.0 months). The association persisted after adjustment for IMDC score, clear vs non-clear cell histology, and prior ICB, with PFS hazard ratio [HR] 0.41 (95% confidence interval [CI], 0.18-0.92, p=0.03) and OS HR 0.21 (95% CI, 0.06-0.79, p=0.02). In exploratory subgroup analyses, the direction of association between high dietary fiber and longer PFS remained consistent independent of treatment with ipilimumab plus nivolumab (ipi/nivo; HR 0.46, 95% CI 0.11-1.94) vs ICB plus VEGF tyrosine kinase inhibitor (HR 0.30, 95% CI 0.09-1.02). Fecal metagenomes from a cohort with RCC and other solid tumors receiving ICB (n = 147) demonstrated associations of PFS with abundance of microbial genes in the pathway fermenting acetyl-CoA into butyrate (p=0.035), a common pathway for microbial fermentation of fiber. We validated our findings using baseline stool sequencing data from an RCC trial of ipi/nivo +/- probiotic CBM-588 (Dizman et al, Nat Med 2022), wherein capacity for butyrate production (i.e., acetyl-CoA pathway gene abundance) was associated with response (p=0.0021) and longer PFS (HR 0.73, 95% CI 0.56-0.96, p=0.022). These findings suggest a taxon-agnostic metabolic functional link between fecal microbiome composition and ICB efficacy and highlight potential for improvements in immunotherapy through microbiome modification.

# Abstract: Molecular characteristics associated with papillary renal cell carcinoma tumor samples according to their diagnostic staging TNM classification

**LEAD AUTHOR**

Raygoza Garay, Juan Antonio, Holden Comprehansive Cancer Center, University of Iowa

**CO-AUTHORS**

Samragnyi Madala, University of Iowa Hospitals and Clinics

Matthew Kroll, University of Iowa Hospitals and Clinics

Sumati Gupta, Huntsman Cancer Institute Laura Graham, University of Colorado Anchutz Medical Campus

Eric Kauffman, Roswell Park Comprehensive Cancer Center

Daniel Grass, Moffitt Cancer Center Jennifer King, Indiana University School of Medicine

Zin Myint, Markey Cancer Center

Paul Viscuse, University of Virginia

Saum Ghodoussipour, Rutgers Cancer Institute Sean Kern, Murtha Cancer Center

Adanma Ayanambakkam, Stephenson Cancer Center

Bodour Salhia, Keck School of Medicine

Michelle Churchman, Aster Insights

Yousef Zakharia, Mayo Clinic

Background. Papillary renal cell carcinoma (pRCC) is a rare subtype of renal cell carcinoma that arises from the renal tubular epithelium and exhibits a papillary growth pattern. Little is known about the molecular characteristics determining the clinical outcomes of patients with this cancer type. We examine how gene expression differs between individuals grouped based on their clinical and pathological TNM staging classification.

Methods. Molecular and clinical data of the patients were obtained as part of the Oncology Research Information Exchange Network (ORIEN) collaboration. Using whole RNA sequencing of primary diagnostic kidney tumor tissue from 105 patients. Sample batch correction was performed to remove technical sources of variation, and the vst normalized expression values were used in subsequent analyses. We defined group membership based on the TNM staging of diagnostic tumor tissue samples into “lower stage” and “higher stage”. Higher stage was defined d as those samples having either a Clinical >T2, or M1 stage, or having either a pathological >T2, >N0, or M1 stage. All the analyses were performed using DESEq2 in the R statistical environment. The “lower stage” group served as the reference for comparison.

Results. The cohort consisted of 105 tumor samples with diagnostic RNASeq. 82% of individuals were male. The individuals had a median age of 65years (IQR 58-69). Based on the classification described above, 75 samples were categorized as “lower stage”, and 26 as “higher stage”. A total of 1826 genes were differentially expressed between the two groups (fdr<0.05 & abs(log2Fc)>2.0. Using the differentially expressed genes, we performed gene set enrichment on the Molecular Signature Database gene sets. We found the following gene sets to be upregulated in the “higher stage” group: HALLMARK_APOPTOSIS (CAV1), HALLMARK_HYPOXIA (SRPX, CAV1), HALLMARK_IL2_STAT5_SIGNALING (RGS16, ADAM19), HALLMARK_UV_RESPONSE_DN (CAV1, MMP16). SRPX, RGS16, and CAV1 have been previously identified as promoting tumorigenesis by inhibiting apoptosis. Pathways associated with immune function were also found to be upregulated in the “higher stage” group: Natural killer cell-mediated cytotoxicity (GZMB, KLRC3, KIR2DL4, KIR2DL1, KIR3DL2), Macrophage-associated genes (FN1, TAC1, LINC00460), among other immune-related pathways.

Conclusion. This study represents a significant step in the molecular understanding of a rare cancer such as pRCC and highlights the strength of collaboration made possible by the ORIEN network. Moreover, it also shows the results that can be obtained with the use of real-world clinical data in conjunction with molecular data. We hope this helps improve the quality of treatment for patients of pRCC.



# Abstract: Mitochondrial health as a biomarker of T cell fitness

**LEAD AUTHOR**

Lindau, Paul, Vanderbilt

**CO-AUTHORS**

Alex Nesta, Vindhya Data Science

Caroline Roe, University of Colorado

Maddelyn Landis, Vanderbilt

Allison Blount, Vanderbilt

Zaid Hatem, Vanderbilt

Jackie Bade, Vanderbilt

Paulo Amaral, Vanderbilt

Anupama Reddy, Vindhya Data Science

Brian Rini, Vanderbilt

Jonatahan Irish, University of Colorado

Jeffrey Rathmell, University of Chicago

Kathryn Beckermann, Tennessee Oncology

Abstract: Checkpoint inhibitors have transformed cancer treatment, yet predicting response remains challenging. T cell mitochondrial function as measured by membrane potential may help delineate functional T cell subsets relevant to anti-tumor immunity.

Methods: Peripheral blood samples were collected from patients with renal cell carcinoma (RCC) and lung cancer undergoing treatment with checkpoint inhibition, as well as from healthy donors. Peripheral blood mononuclear cells (PBMC) were isolated and stained for mitochondrial membrane potential using tetramethylrhodamine ethyl ester (TMRE) staining. T cell subsets and function were assessed using mass cytometry, single-cell RNA sequencing, as well as TCR analysis.

Results: Cells sorted for high mitochondrial membrane potential (TMRE high) displayed a more metabolically activated state, with increased expression of metabolic regulators such as CPT1a and Cytochrome C. Therefore, TMRE high cells were called mitochondrially active, while TMRE low cells will be referred to as mitochondrial poised cells, reflecting a lower level of metabolic activation despite preserved with preserved effector potential.

Mitochondrially poised CD8+ T cells exhibited high Granzyme B expression, indicating a cytotoxic phenotype with immediate effector functionality. In responders compared to non-responders, mitochondrially poised cells cells demonstrated features of an effector memory-like state, with low metabolic energy requirements and showed an enrichment for TCRs matching tumor-infiltrating lymphocytes (TILs), underscoring their role in immediate anti-tumor cytotoxicity. In contrast, mitochondrially active T cells (TMRE high) were enriched for memory-like subsets with terminally differentiated and regulatory phenotypes within both CD4+ and CD8+ compartments, suggesting a role in sustaining long-term immune responses rather than immediate cytotoxicity.

Conclusions: Mitochondrially poised T cells resemble effector memory subsets, are enriched for TIL-matched TCRs, and are associated with immediate anti-tumor cytotoxicity. In contrast, mitochondrially active T cells show high metabolic activation and features of terminal differentiation and regulatory potential. These findings highlight T cell mitochondrial function as a potential marker for predicting T cell functionality and response to immunotherapy. ne

# Abstract: Prognostic Signatures Associated with TP53 mutations in chromophobe renal cell carcinoma

**LEAD AUTHOR**

Maulding, Nathan, Tempus AI, Inc

**CO-AUTHORS**

Andrew Sedgewick, Tempus AI, Inc

Elizabeth Ellis, MD Anderson

Niki Zacharias, MD Anderson

Justin Guinney, Tempus AI, Inc

Pavlos Msaouel, MD Anderson

Background: In chromophobe renal cell carcinoma (chRCC), TP53 mutations correlate with advanced disease and chromosomal instability, but the downstream gene expression programs and potential therapeutic vulnerabilities remain unclear.

Methods: We analyzed de-identified clinical data, targeted DNA sequencing, and bulk RNA-seq from 168 chRCC specimens and 226,734 tumors across 25 additional cancer cohorts in the Tempus database. Because chromosomal instability (CIN) is a hallmark consequence of TP53 loss, we applied the 67-gene CINSARC signature, originally developed to quantify CIN risk across solid tumors, to determine whether TP53 status predicts global CIN activity in chRCC. TRIP13, a spindle-checkpoint ATPase included in CINSARC and repeatedly implicated in CIN tolerance, was pre-specified for individual analysis as a tractable, mechanistically relevant candidate target. To evaluate pathway activity, we used GSEA to calculate normalized enrichment scores (NES) between groups.

Results: Tumors with TP53 mutations had significantly higher expression of TRIP13 (p < 1 × 10^-10^), and enriched CINSARC pathway activity. Other pathways enriched for TP53MUT included G2-M Checkpoint (NES = 2.26, FDR < 0.001), E2F Targets (NES = 2.16, FDR < 0.001) and Mitotic Spindle (NES = 2.10, FDR < 0.001). TRIP13 was highly correlated with mean CINSARC expression (r=0.92, p < 1e-65), and both markers showed strong association with Stage 4 tumors (p < 1 × 10^-3^) and distant metastases (p < 1 × 10^-4^). Other cancer types also showed association between TP53MUT and TRIP13 or CINSARC including Thyroid, Liver, Soft tissue sarcoma, and Breast (p < 1 × 10-50).

Conclusions: CINSARC effectively captures the TP53-dependent CIN phenotype in chRCC, and TRIP13 emerges as its dominant, potentially targetable effector. Together, TRIP13 and CINSARC serve as progression biomarkers and nominate TRIP13 inhibition as a rational therapeutic strategy that merits further investigation for advanced chRCC and other p53-deficient malignancies.



# Abstract: NPRL2 loss silences STING signaling from genomic instability and promotes a CD8lowM2high microenvironment

**LEAD AUTHOR**

Liu, Xiande, The University of Texas MD Anderson Cancer Center

**CO-AUTHORS**

Muhammed Onel, The University of Texas MD Anderson Cancer Center

Hongchao He, The University of Texas MD Anderson Cancer Center

Xuesong Zhang, The University of Texas MD Anderson Cancer Center

Yanting Zhang, The University of Texas MD Anderson Cancer Center

Tatiana Karpinets, The University of Texas MD Anderson Cancer Center

Kai Jiang, The University of Texas MD Anderson Cancer Center

Anh Hoang, The University of Texas MD Anderson Cancer Center

Christine Peterson, The University of Texas MD Anderson Cancer Center

Jianhua Zhang, The University of Texas MD Anderson Cancer Center

Guang Peng, The University of Texas MD Anderson Cancer Center

Eric Jonasch, The University of Texas MD Anderson Cancer Center

NPRL2 is located on chromosome 3p, and its deep deletion and reduced expression were identified in ccRCC. However, its influences on anti-tumor immunity and immunotherapy response remain unclear.

The lysosome plays important roles in DNA repair and cytosolic DNA degradation. We found that NPRL2 loss promoted the phosphorylation of TFEB and prevented its translocation to the nucleus, indicating impaired lysosome biogenesis. NPRL2 deficiency, TFEB deficiency and pharmacologic lysosomal inhibition activated the ATM-CHK2 DNA damage response pathway and led to the accumulation of cytosolic DNA, while TFEB activation with ML-SA1 yielded the opposite results. Unexpectedly, the increased cytosolic DNA in NPRL2 knockout cells failed to activate, and instead, suppressed the cGAS-STING pathway. We further found that NPRL2 loss suppressed the expression of STING at both mRNA and protein levels. By analyzing the KIRC TCGA dataset, we found that low NPRL2 expression was associated with reduced CD8 T cell infiltration but increased M2 macrophages infiltration, which were clustered with increased expression of CD274, PDCD1LG2, PVR, NECTIN2, and CD163. Similarly, Nprl2 knockdown Renca tumors demonstrated paucity of T cells and abundance of M2 macrophages. Nprl2 loss reduced the complete response rate to anti-PD1 therapy in a syngeneic tumor model. The ATM inhibitor KU60019 increased T cell infiltration but showed no evidence of synergistic effect with anti-PD1 therapy, indicating the presence of additional immunosuppressive pathways other than PD1-PDL1/L2 axis.

These results indicate that NPRL2 loss regulates DNA damage and cytosolic DNA accumulation via lysosomal inhibition, and on the other hand, silences cytosolic DNA response by suppressing STING expression. NPRL2 loss defines a genomically unstable but M2 macrophage dominant tumor phenotype associated with checkpoint inhibitor resistance in RCC.

Acknowledgement: This project was supported by KCA grant awarded to Drs. Jonasch and Peng.



# Abstract: AI-Driven Multiplex Immunofluorescence Analysis of the Tumor Immune Microenvironment to Predict Immunotherapy Response in Clear Cell Renal Cell Carcinoma

**LEAD AUTHOR**

Yang, Yuanquan, Division of Medical Oncology, Department of Internal Medicine, The Ohio State University Comprehensive Cancer Center, Columbus, OH, USA

**CO-AUTHORS**

Rajeev Jaundoo, Steve and Cindy Rasmussen Institute for Genomic Medicine, Nationwide Children’s Hospital, Columbus, OH, USA

Mingjia Li, Division of Medical Oncology, Department of Internal Medicine, The Ohio State University Comprehensive Cancer Center, Columbus, OH, USA

Jordan Krull, Department of Biomedical Informatics, The Ohio State University Comprehensive Cancer Center, Columbus, OH, USA

Qin Ma, Department of Biomedical Informatics, The Ohio State University Comprehensive Cancer Center, Columbus, OH, USA

Lingbin Meng, Division of Medical Oncology, Department of Internal Medicine, The Ohio State University Comprehensive Cancer Center, Columbus, OH, USA

Amir Mortazavi, Division of Medical Oncology, Department of Internal Medicine, The Ohio State University Comprehensive Cancer Center, Columbus, OH, USA

Yoshinobu Koguchi, Pelotonia Institute for Immuno-Oncology, The Ohio State University, Columbus, OH, 43210

Mark Rubinstein, Pelotonia Institute for Immuno-Oncology, The Ohio State University, Columbus, OH, 43210

Swati Satturwar, Department of Pathology, The Ohio State University Comprehensive Cancer Center, Columbus, OH, USA

Anil Parwani, Department of Pathology, The Ohio State University Comprehensive Cancer Center, Columbus, OH, USA

Eric A. Singer, Division of Urologic Oncology, The Ohio State University Comprehensive Cancer Center, Columbus, OH, USA

Zihai Li, Pelotonia Institute for Immuno-Oncology, The Ohio State University, Columbus, OH, 43210

W. Kim Rathmell, Division of Medical Oncology, Department of Internal Medicine, The Ohio State University Comprehensive Cancer Center, Columbus, OH, USA

Jayajit Das, Steve and Cindy Rasmussen Institute for Genomic Medicine, Nationwide Children’s Hospital, Columbus, OH, USA

Background: Predicting immune checkpoint inhibitor (ICI) response in clear cell renal cell carcinoma (ccRCC) remains challenging due to the heterogeneous tumor immune microenvironment (TIME). We hypothesize that a vision-language artificial intelligence (AI) model can identify microscopic spatial patterns (MSPs) within TIME that correlate with ICI response.

Methods: Primary tumors from metastatic ccRCC patients treated with ICI at Ohio State (2008–2019) were analyzed using the Vectra Polaris™ imaging system. Whole-slide multiplex immunofluorescence (mIF) images were generated using T-cell (CD3, CD8, PD1, FoxP3, GzB, Tbet) and myeloid (CD68, CD86, CD163, CD11b, CD11c, panCK) panels. The Prov-GigaPath AI foundation model produced image embeddings representing MSPs, which were used to train and test machine learning (ML) classifiers. Balanced accuracy assessed model performance.

Results: Ninety mIF whole-slide images from 45 patients (median age 61; 68% male) were analyzed. ICI regimens included nivolumab (49%), nivolumab + ipilimumab (42%), and ICI + TKI (7%); 49% received ICI as first-line therapy. Based on RECIST 1.1, 20 patients were responders (CR+PR) and 25 were nonresponders (SD+PD). Patients were randomly split into 50 training/testing sets (3:1 ratio). The top-performing model was a support vector classifier (SVC) using myeloid panel embeddings (average accuracy 62%), followed by logistic regression (61%), k-nearest neighbors (59%), random forest (55%), and decision tree (51%). Models using T-cell panel embeddings performed less well (top SVC accuracy 56%).

Conclusion: Prov-GigaPath combined with SVC shows promise for predicting ICI response in ccRCC. Future work will focus on improving model accuracy, interpretability, and external validation.
